# Dissecting neural correlates of theory of mind and executive functions in behavioral variant frontotemporal dementia

**DOI:** 10.1186/s13195-024-01596-4

**Published:** 2024-10-26

**Authors:** Christopher M. Weise, Annerose Engel, Maryna Polyakova, Qiong Wu, Karsten Mueller, Sabine Herzig, Robert Jech, Janine Diehl-Schmid, Lina Riedl, Sarah Anderl-Straub, Johannes Kornhuber, Klaus Fassbender, Jens Wiltfang, Klaus Fliessbach, Johannes Prudlo, Matthis Synofzik, Adrian Danek, Markus Otto, Matthias L. Schroeter, for the FTLD Consortium Germany, Maryna Polyakova, Janine Diehl-Schmid, Lina Riedl, Sarah Anderl-Straub, Johannes Kornhuber, Klaus Fassbender, Jens Wiltfang, Klaus Fliessbach, Johannes Prudlo, Matthis Synofzik, Adrian Danek, Markus Otto, Matthias L. Schroeter, Fabiola Böhm, Marie Söntgerath, Lea Hüper, Anke Marschhauser, Danièle Pino, Frank Regenbrecht, Angelika Thöne-Otto, Carola Roßmeier, Leonie Werner, Juan Manuel Maler, Tanja Richter-Schmidinger, Anke Hammer-Kaspereit, Timo Oberstein

**Affiliations:** 1 Department of Neurology, Halle University Medical Center, Halle (Saale), Germany; 2 Department of Neurology, Max Planck Institute for Human Cognitive and Brain Sciences, Leipzig, Germany; 3 Clinic for Cognitive Neurology, University of Leipzig Medical Center, Leipzig, Germany; 4 Department of Neurology, Charles University in Prague, 1st Faculty of Medicine and General University Hospital in Prague, Prague, Czech Republic; 5 Department of Psychiatry and Psychotherapy, Technical University of Munich, School of Medicine, Munich, Germany; 6 Clinical Center for Psychiatry, Psychotherapy, Psychosomatic Medicine, Geriatrics and Neurology, kbo-Inn-Salzach-Klinikum, Wasserburg/Inn, Germany; 7 Department of Neurology, University of Ulm, Ulm, Germany; 8 Department of Psychiatry and Psychotherapy, University of Friedrich-Alexander-Erlangen, Erlangen, Germany; 9 Department of Neurology, Saarland University Hospital Homburg, Homburg, Germany; 10 Department of Psychiatry and Psychotherapy, University of Göttingen, Göttingen, Germany; 11 German Center for Neurodegenerative Diseases (DZNE), Bonn, Germany; 12 Department of Neurodegenerative Diseases and Geriatric Psychiatry, University Hospital Bonn, Bonn, Germany; 13 Department of Neurology, Rostock University Medical Center, Rostock, Germany; 14 Department of Neurodegenerative Diseases, Center of Neurology, Hertie Institute for Clinical Brain Research, Tübingen, Germany; 15 Department of Neurology, Ludwig-Maximilians-University Munich, München, Germany

**Keywords:** Executive functions, Frontotemporal dementia, Magnetic resonance imaging, Reading the Mind in the Eyes Test, Social cognition, Theory of mind

## Abstract

Behavioral variant frontotemporal dementia (bvFTD) is characterized by profound and early deficits in social cognition (SC) and executive functions (EF). To date it remains unclear whether deficits of the respective cognitive domains are based on the degeneration of distinct brain regions. In 103 patients with a diagnosis of bvFTD (possible/probable/definite: *N* = 40/58/5) from the frontotemporal lobar degeneration (FTLD) consortium Germany cohort (age 62.5±9.4 years, gender 38 female/65 male) we applied multimodal structural imaging, i.e. voxel-based morphometry, cortical thickness (CTH) and networks of structural covariance via source based morphometry. We cross-sectionally investigated associations with performance in a modified Reading the Mind in the Eyes Test (RMET; reflective of theory of mind - ToM) and five different tests reflective of EF (i.e. Hamasch-Five-Point Test, semantic and phonemic Fluency, Trail Making Test, Stroop interference). Finally, we investigated the conjunction of RMET correlates with functional networks commonly associated with SC respectively ToM and EF as extracted meta-analytically within the Neurosynth database. RMET performance was mainly associated with gray matter volume (GMV) and CTH within temporal and insular cortical regions and less within the prefrontal cortex (PFC), whereas EF performance was mainly associated with prefrontal regions (GMV and CTH). Overlap of RMET and EF associations was primarily located within the insula, adjacent subcortical structures (i.e. putamen) and the dorsolateral PFC (dlPFC). These patterns were more pronounced after adjustment for the respective other cognitive domain. Corroborative results were obtained in analyses of structural covariance networks. Overlap of RMET with meta-analytically extracted functional networks commonly associated with SC, ToM and EF was again primarily located within the temporal and insular region and the dlPFC. In addition, on a meta-analytical level, strong associations were found for temporal cortical RMET correlates with SC and ToM in particular. These data indicate a temporo-frontal dissociation of bvFTD related disturbances of ToM and EF, with atrophy of the anterior temporal lobe being critically involved in ToM deficits. The consistent overlap within the insular cortex may be attributable to the multimodal and integrative role of this region in socioemotional and cognitive processing.

**Supplementary Information:**

The online version contains supplementary material available at 10.1186/s13195-024-01596-4.

## Introduction

Frontotemporal lobar degeneration (FTLD) comprises a variety of pathologically and clinically heterogenous neurodegenerative diseases. Although common aspects include atrophy of the frontal and temporal lobes, the clinical spectrum ranges from language impairments to profound behavioral disturbances, depending on the initial site of affection. Based on predominant clinical features FTLD can be clinically classified as primary progressive aphasia (PPA) and its subtypes, and behavioral variant frontotemporal dementia (bvFTD) with the latter being the most frequent form of FTLD. According to the International Consensus Criteria for bvFTD [1] early symptoms include behavioral disinhibition, apathy or inertia, loss of sympathy or empathy, perseverative, stereotyped, compulsive or ritualistic behavior, hyperorality and dietary changes, as well as deficits in executive functions (EF). Generally, deficits of EF are considered a typical and early clinical manifestation of bvFTD [2, 3], although preserved EF may occur in a subset of bvFTD patients [4–8]. Additionally, broad evidence points to profound deficits of social cognition (SC). Consequently, SC deficits have been included as a further criterion for bvFTD in the latest edition of the Diagnostic and Statistical Manual of Mental Disorders (i.e. DSM-5, 2013) [9]. Importantly, tests of SC have been found to provide higher discriminative power as compared to tests of EF with respect to classifying bvFTD patients vs. healthy controls [10].

EF comprise a number of cognitive processes related to flexible, motivated and goal directed behavior with cognitive flexibility, inhibitory control and working memory being considered core EFs [11]. Social cognition, on the other hand, describes a variety of cognitive capabilities that enable contextually and socially adequate behavior and interactions with other humans. As a key aspect of social cognition, theory of mind (ToM) - or mentalizing - allows humans to identify and value emotional and mental states of other people (e.g. review in Happé et al. [12]). Nevertheless, social interactions as complex aspect of human behavior require a high degree of cognitive control, flexibility and variability [13], thus naturally demanding the integration and interaction of SC with other cognitive processes and EF in particular. Moreover, developmental studies point to a complex relationship and co-dependency of EF with SC and ToM including overlap of its neurobiological foundations [14].

A large number of studies has investigated the neurobiological underpinnings of SC and ToM in healthy humans. Meta-analyses of functional neuroimaging studies most consistently found engagement of the temporal cortex including the temporo-parietal junction (TPJ), frontoinsular and medial prefrontal cortical regions [15–17]. Considering neurodegenerative diseases and bvFTD in particular, several studies – however mostly with smaller sample sizes - have investigated the neural correlates of SC and ToM deficits [8, 18–26]. Overall, these studies most commonly found prefrontal, temporal and insular structures to be related with different aspects of SC.

In this study we aimed to explore the relationship of ToM deficits with brain structural parameters in a large and well characterized sample of participants with a diagnosis of bvFTD from the German FTLD consortium [27, 28]. Since SC and EF are regarded as the main cognitive domains affected by bvFTD and codependent, we specifically aimed to investigate the brain structural correlates of EF deficits and potential overlapping and/or independent associations of the respective cognitive domain’s deficits in order to disentangle the intricate relationship of EF and SC deficits in bvFTD. To do so we applied multi-parametric structural imaging analyses including voxel-based morphometry (VBM) and cortical thickness (CTH) to investigate associations of ToM (as one key aspect of SC) and several tests reflective of EFs. We opted to apply the above-mentioned imaging methods as they provide complementary but not identical information regarding brain structural alterations which may be of particular value when used in combination. In addition to VBM and CTH based structural associations across the whole brain we aimed to interpret and embed our results on a superordinate level. To do so we additionally investigated gray matter structural covariance networks as derived by source-based morphometry and by validating our results via conjunction or overlap analyses with brain regions commonly activated in the context of SC, ToM and EF [15] as previously described [2, 29, 30]. We hypothesized that impairments in ToM deficits in bvFTD are related to temporal, insular and medial prefrontal cortical regions [15–17], at least partially overlapping with the neural correlates of EF mainly associated with prefrontal regions [3].

## Materials and methods

### Participants

All cognitive and structural imaging data were provided by the multi-centric FTLD consortium’s study Germany [28]. The entire study population included 103 participants from ten study sites (i.e. Bonn, Erlangen, Goettingen, Homburg, Leipzig, Munich LMU, Munich TU, Rostock, Tuebingen, Ulm) with a diagnosis of possible, probable according to Rascovsky et al. [1] or definite bvFTD (via genetic testing) and available structural imaging and cognitive data. For detailed information regarding the study’s protocol, we refer to Otto et al. [28]. In brief, all participants underwent a standardized battery of clinical and neuropsychological assessments, as well as neuroimaging and cerebrospinal fluid biomarker investigations. Note that the study applied strict standard operating procedures (SOPs).

### Neuropsychological and cognitive testing

Neuropsychological and behavioral tests have been applied as previously described in Schroeter et al. [10]. Please note differences of data availability as detailed in Table 1. In brief, tests included – among others – the FTLD Clinical Dementia Rating scale (FTLD-CDR), which in in contrast to the original CDR (range 0–18) also considers behavior/personality and language, extending its sensitivity to other dementia syndromes beyond Alzheimer’s dementia [31] (range 0–24). International Standard Classification of Education (ISCED) was used to range educational level into seven levels [32]. Behavioral aspects were assessed via the modified Frontal Systems Behavior Scale (FrSBe; range 24–120; subscales include apathy, disinhibition, executive dysfunction; here companion ratings are reported) [33]. Apathy was quantified via the Apathy Evaluation Scale (AES) (range 0–54; here companion ratings are reported) [34].

**Table 1:** Population characteristics

**Parameter**	**Mean ± SD/frequency**	**Data availability**
Age in years (*N*=103)	62.6 ± 9.4	100% (*N*=103)
Gender (female/male; number)^*^	38/65	100% (*N*=103)
Possible/Probable/Definite bvFTD (number)^*^	40/58/5	100% (*N*=103)
Disease duration in years	4.0 ± 4.5	97% (*N*=100)
Education in years	13.6 ± 2.9	98% (*N*=101)
ISCED level (0/1/2/3/4/5/6; number)^*^	0/1/17/39/19/21/6	100% (*N*=103)
CDR (0 to 18)	5.6 ± 3.4	91% (*N*=94)
FTLD CDR (0 to 24)	7.7 ± 4.3	91% (*N*=94)
AES^C^ (0 to 54)	34.3±10.4	76% (*N*=78)
FrSBe^C^ total frequency (24 to 120)	72.5 ± 17.6	73% (*N*=75)
FrSBe^C^ total distress (24 to 120)	64.2 ± 18.1	64% (*N*=66)
MMSE (0 to 30)	24.5 ± 5.0	95% (*N*=98)
RMET (number of correct items)	11.1 ± 3.5	78% (*N*=80)
H5PT (number of correct patterns)	16.0 ± 9.7	86% (*N*=89)
TMT B/A ratio	3.0 ± 1.3	62% (*N*=64)
sFLU (items reported in 60 s)	12.2 ± 6.8	92% (*N*=95)
pFLU (items reported in 60 s)	7.2 ± 5.0	89% (*N*=92)
Stroop (incongruent / color naming ratio)	0.43 ± 0.19	70% (*N*=72)

All data are shown as mean ± standard deviation (SD) except for ^*^; ^C^ rated by companion

*ISCED *International Standard Classification of Education, *CDR* Clinical Dementia Rating, *FTLD CDR *Frontotemporal Lobar Degeneration-Modified Clinical Dementia Rating, *AES* Apathy Evaluation Scale, *FrSBe *Frontal Systems Behavior Scale, *MMSE* Minimental State Examination, *RMET* Reading the Mind in the Eyes Test, *H5PT *Hamasch-Five- Point Test, *TMT *Trail Making Test B/A, *sFLU* semantic Fluency, *pFLU *phonemic Fluency

Mentalizing (i.e. ToM) was assessed via a modified Reading the Mind in the Eyes Test (RMET) [35], which covers both cognitive and affective aspects of ToM (for more details see [10]). Here, participants were asked to choose among four predetermined categories to describe the emotional or mental state of a total of 24 photographs of the human eye region. The resulting score is based on the number of correct answers with higher scores indicating better performance. This test measures an individual’s capability to identify emotional and mental states based on the eye region, which is considered a key aspect of SC.

EFs were assessed via five different neuropsychological tests as previously described [10].

*Hamasch-Five-Point Test* (H5PT) was used to measure figural fluency [36] which reflects both processing speed and cognitive flexibility. Here, participants are asked to connect five points with the goal to generate as many different patterns as possible within a given time of three minutes. The score is based on the number of correct patterns with higher scores indicating better performance.

Cognitive flexibility and speech abilities were assessed with two different forms of verbal fluency tasks [37–39]. For the *semantic Fluency* task (sFLU), participants were asked to name as many words as possible within a given category (i.e. “animals”) and time. This test additionally captures semantic memory. *Phonemic Fluency* (pFLU), on the other hand, was assessed by the number of words beginning with the letter “S”. Both scores consist of the number of correct words that have been generated within 60 s, with higher scores indicating better performance.

The *Trail Making Test* (TMT) is widely used as another index of EF. It consists of two parts: In Part A, participants are asked to connect numbers only (1 to 25), while in Part B both numbers and letters are presented (i.e. 1 to 13 and A to L) and have to be connected alternately (i.e. 1 – A – 2 – B etc.). Compared to Part A, Part B is more dependent on cognitive flexibility and selective attention. The TMT B/A ratio for completion has been found to be a superior measure of EF [40], which has therefore been used in our study. Of note, for the TMT B/A ratio higher values indicate worse performance.

The *Stroop* color-word interference task is performed by urging participants to name the color of a word with an incongruent meaning (e.g. the word “blue” written in red), thus requiring the inhibition of an overlearned response (i.e. reading) for the benefit of a less common response (i.e. color naming). This test has been shown to reliably measure interference resolution and response inhibition. Here performance is scored via the ratio of correct answers in 45 s as described above and the correct answers when the participants were asked to name the color only [41–43].

### Imaging procedures

Structural magnetic resonance imaging (MRI) (i.e. T1-MPRAGE-images; High resolution Magnetization-Prepared Rapid Gradient Echo) was performed on Siemens Magnetom 3T scanners (Verio, Skyra, Trio, Allegra, Biograph mMR; Erlangen, Germany) or a GE Signa HDxt scanner. Detailed scanner parameters have been previously reported (please see supplementary data in Chapman et al.) [44].

SPM12 (http://www.fil.ion.ucl.ac.uk/spm) and the CAT12 toolbox (https://neuro-jena.github.io/cat/) were used for structural image preprocessing and analyses as previously described [45]. In brief, preprocessing of VBM data was performed with default settings of the CAT12 pipeline and included bias field corrections, tissue segmentation (i.e. gray matter, white matter, cerebrospinal fluid) followed by spatial normalization to the DARTEL template within the Montreal Neurological Institute (MNI) space and with an isotropic voxel size of 1.5 mm. Next, gray matter (GM) data were smoothed with an 8 mm full-width-half-maximum (FWHM) isotropic Gaussian kernel.

Preprocessing of surface based CTH data was performed via the projection-based thickness method as implemented in CAT12 [46], which includes estimation of CTH and reconstruction of the bilateral hemispheric central surface. CTH preprocessing comprises partial volume correction as well as correction for sulcal blurring and asymmetries respectively. As final step, reconstructed left and right hemispheres with the corresponding CTH data were merged and smoothed with a 15 mm FWHM Gaussian kernel. Quality control was performed by visually checking preprocessed GM and CTH data and by using automated measures of quality control of the CAT12 toolbox (i.e. sample homogeneity analyses). Quality control revealed some outliers, nevertheless, upon close review we rated these as disease associated due to profound cerebral atrophy, thus not excluding these participants from our analyses. Additional sensitivity analyses were performed to explore scanner effects. Here, after including scanner sites via nine separate dummy variables showed highly comparable, yet partly less extent patterns as compared to the original analyses without correction for scanner sites. Since this was deemed more attributable to an artificial reduction of variance (and degrees of freedom) then to a true scanner effect we chose to present results uncorrected for scanner site.

### Statistical analyses

#### Hypothesis-driven approaches

Multiple regression analyses as implemented in SPM12 and CAT12 were used to analyze the relationship of RMET and EF measures (i.e. H5PT, sFLU, pFLU, TMT, Stroop) with VBM based GM volume (GMV) and surface based CTH. All models included age and gender as covariates. For analyses of VBM data total intracranial volume (TIV) was additionally included as covariate. Additionally an absolute threshold mask of 0.1 was applied (thus excluding voxels below a GM value of 0.1) in order to reduce the risk of artifacts. Analyses of CTH data, on the other hand, do not require correction for TIV or absolute threshold masking.

In addition, we investigated independent associations of RMET and measures of EF. Here, generalized linear models with all variables of interest were used to identify measures of EF being independently associated with RMET. Next, associations of RMET and EF measures with GMV and CTH were investigated by additionally correcting for the other respective variable (e.g., in the investigation of the relationship between RMET and GMV, the analysis was adjusted to account for the potential influence of EF).

Statistical significance was determined with the Threshold-Free Cluster Enhancement (TFCE) toolbox (dbm.neuro.uni-jena.de). TFCE uses permutation-based, non-parametric statistics that consider both local extent and intensity without having to predetermine arbitrary thresholds [47]. Here, analyses were performed with 5000 permutations. Although GMV and CTH as well as measures of EF are codependent and cannot be considered entirely independent we chose to set significance conservatively at *p* < 0.005 FWE_TFCE_ whole brain corrected in order to additionally account for multiple testing. In our overlap and post-hoc analyses (i.e. independent associations of RMET and H5PT) we also employed a more liberal threshold (*p* < 0.05 FWE_TFCE_) for explorative purposes.

VBM results were illustrated with the MRIcron Software. (https://people.cas.sc.edu/rorden/mricron/index.html). Overlap images of VBM data for conjunction analyses were created with binarized maps of the original results. CTH results were illustrated with CAT12’s in-built surface overlay option. For anatomical labeling, we primarily used the MNI2Tal online tool (https://bioimagesuiteweb.github.io/bisweb-manual/tools/mni2tal.html). Additional labeling was done with the Talairach Client (http://www.talairach.org/client.html) in order to provide further in depth anatomical information (when reasonable applicable).

#### Data-driven approaches

Beside our hypothesis-driven approaches, we wanted to validate our results with independent procedures. As additional data-driven approach, we applied source-based morphometry (SBM) as implemented in the Group ICA of functional (f)MRI Toolbox (GIFT version 4.0; http://mialab.mrn.org/software/gift/). Detailed descriptions of this method can be found in Xu et al. [48]. Source-based morphometry allows the identification of multiple covarying brain regions (i.e. “structural covariance networks”) based on brain structural MRI data such as VBM based GMV [48, 49]. For our study, preprocessed VBM data of all *N* = 103 subjects were subjected to the SBM analysis pipeline with default settings. Prior to the actual analysis we chose to evaluate the optimal number of spatially independent components as implemented in the GIFT toolbox via the minimum description length (MDL) principle, which yielded a total number of *N* = 12 independent components (i.e. structural covariance networks; C1 – C12). Next, SBM processing via independent component analyses (i.e. Infomax algorithm) was performed to decompose brain structural data into the predefined number of individual components. This step involves the conversion and decomposition of GM data into a mixing matrix and source matrix. Eventually, this results in subject- and component-wise, z-transformed loading coeffcients, which indicate the contribution of these covariance patterns to each participants individual GM data and can be used for further statistical analyses. Upon review of the components, component 7 appeared as artifact burdened, however, for the sake of completeness we still chose to include this component.

Source-based morphometry derived loading coefficients and non-imaging data were analyzed with SAS statistical software (SAS Institute Inc, version 9.4, Cary, NC). To analyze the correlations of SBM derived structural covariance networks (i.e. C1 – C12) with cognitive test performance we used partial Spearman correlations, adjusted for age and gender, resulting in a total of *N* = 72 individual analyses. To account for multiple comparisons we applied the false discovery rate (*p* < 0.05 FDR; www.sdmproject.com/utilities/?show=FDR) with additional reporting of trend-level significant (*p* < 0.1 FDR) correlations. Illustrations and coordinate data of SBM results are reported with a threshold of z = 2.0.

#### Meta-analytical approaches

To further validate our findings we adopted additional meta-analytical methods by making use of the Neurosynth database (https://neurosynth.org/), similar to a recently described approach.

The Neurosynth database contains a large number of functional imaging studies and allows term-based meta-analyses. For our study we used the terms “social” (1302 studies identified; 47083 activations reported), “theory mind” (181; 7761) and “executive” (786; 28937). Corresponding masks were downloaded and used to investigate the overlap with brain structural correlates of RMET (VBM) in bvFTD. Of note, Neurosynth offers two different types of meta-analyses: associations test maps, which indicate whether activation in a certain region can be observed more consistently in studies mentioning the term of interest (e.g. “executive”) as compared to other studies that do not include the term of interest, whereas uniformity test maps indicate consistent activation across all studies that mention the particular term of interest. For our study, we used both associations and uniformity test maps. Prior to the creation of overlap maps (via MRIcron Software), all statistical maps of positive associations with brain regional connectivity were binarized.

In addition, we made use of location-based Neurosynth meta-analyses. Here, coordinates from significant independent associations (i.e. after corrections for EF and RMET respectively) were entered into the Neurosynth database. For this kind of analysis Neurosynth provides a number of different metrics: (1) Z-scores for the respective locations and their associations with meta-analytical maps for various terms. (2) The posterior probability shows the probability of specifics terms being used (e.g. “theory mind”) in studies that report activations for the location being searched. Location based functional connectivity (r) describes the similarity of seed-based functional connectivity maps based on the entered location with term-based meta-analytic maps (i.e. via Pearson correlation with whole brain reverse-inference maps). Meta-analytic coactivation (r) provides a similar metric but makes use of Neurosynth’s meta-analytic coactivation maps.

## Results

### Demographic and clinical measures

Basic demographic information and detailed information on cognitive performance of our study population is listed in Table 1. *N* = 5 patients were classified as definite bvFTD based on genetic testing (positivity for: C9orf72 *N* = 3; GRN (Progranulin) *N* = 1; BTNL2 *N* = 1). Expectedly, RMET performance covaried significantly with measures of EF, except for Stroop test (all partial Spearman correlations with RMET adjusted for age and gender: H5PT [*N* = 80, *r* = 0.46, *p* < 0.0001]; sFLU [*N* = 77, *r* = 0.49, *p* < 0.0001]; pFlu [75, *r* = 0.49, *p* < 0.0001]; Stroop [*N* = 69, *r* = 0.21, *p* = 0.09]; TMT B/A [*N* = 61, *r* = -0.31, *p* = 0.02]). Generalized linear models showed independent significant associations of both sFLU (*p* = 0.01) and H5PT (*p* = 0.03) with RMET. No significant associations were found for pFLU, Stroop and TMT B/A (all *p* > 0.5). These results remained similar after additional controlling for age and gender. We applied no corrections for multiple comparisons for these models.

### Associations of Reading the Mind in the Eyes and executive functions test performance with gray matter volume

**Fig. 1:**
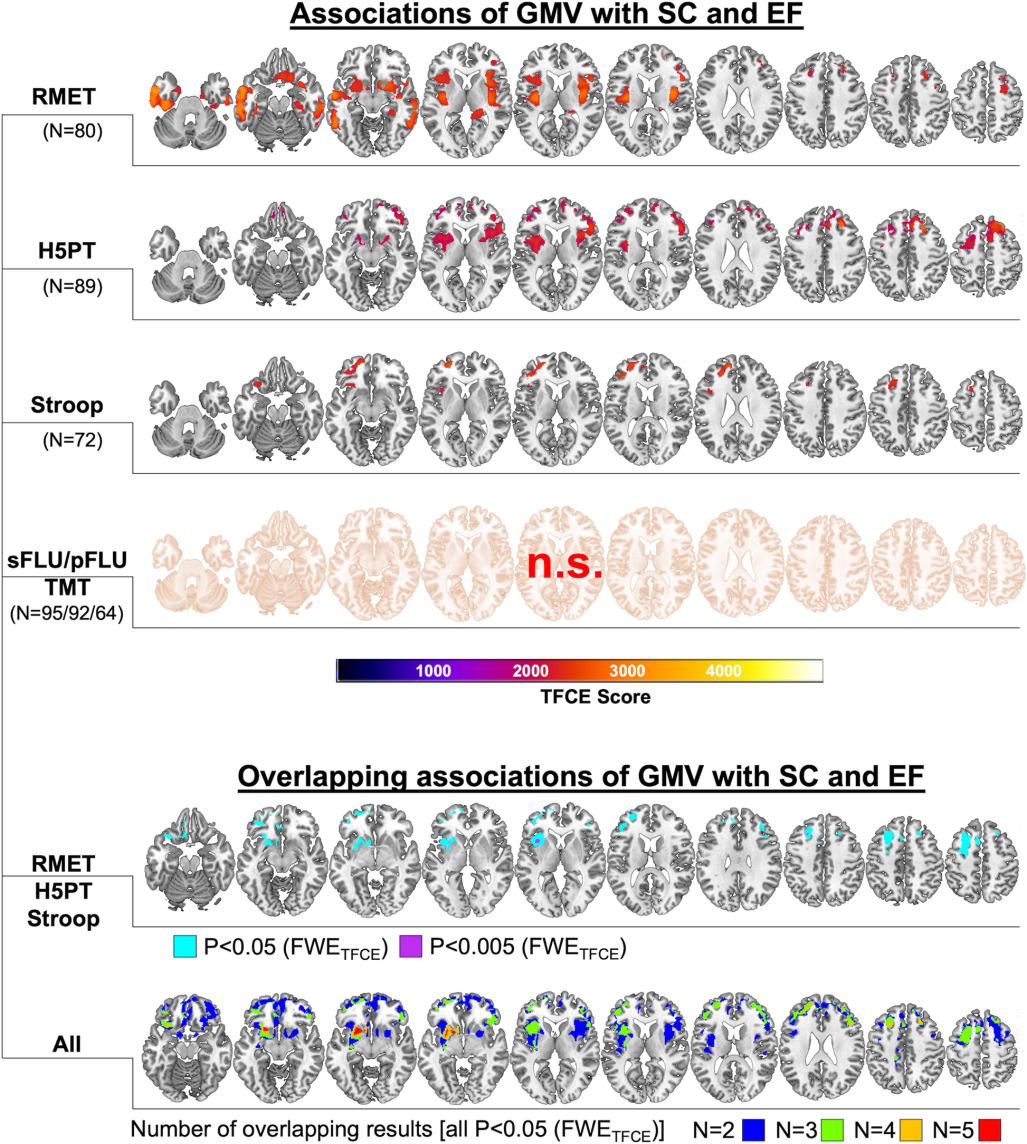
Associations of gray matter volume (GMV) via voxel-based morphometry (VBM) (top four rows) with Reading the Mind in the Eyes Test (RMET) performance as a measure of social cognition (SC) and five different tests reflective of performance in executive functions (EF). Voxel based morphometry (VBM) results are thresholded at *p* < 0.005 FWE_TFCE_. All results indicate positive associations of test performance with GMV with warmer colors indicating stronger associations. The two bottom rows highlight overlapping regions of VBM results [here additional liberal thresholds (i.e. *p*<0.05 FWE_TFCE_) were applied for exploratory purposes]. Please note that colors of the last row’s illustrations are not reflective of individual tests, but highlight the number of overlapping results. Left side of the brain is shown on the left. *RMET* Reading the Mind in the Eyes Test; *H5PT *Hamasch-Five-Point Test, *TMT *Trail Making Test B/A; *sFLU* semantic Fluency; *pFLU *phonemic Fluency

For detailed results, see Fig. 1 and supplementary Table S2. For all analyses (*p* < 0.005 FWE_TFCE_) we only found positive associations for GMV with better test performance or, vice versa, associations between atrophy and worse test performance.

Multiple regression analyses showed widespread positive associations of RMET (*N* = 80) performance with GMV of the bilateral insular cortex and adjacent parts of the basal ganglia (i.e. putamen) and with the bilateral temporal and prefrontal cortex (i.e., right orbitofrontal [OFC] and bilateral dorsolateral [dlPFC] PFC). H5PT (*N* = 89) showed extensive positive associations with prefrontal cortical regions including the bilateral OFC and dlPFC. Additional associations included the insula and basal ganglia (i.e. putamen).

Better Stroop performance (*N* = 72) was associated with more GMV of the left frontoinsular region, as well as left prefrontal cortical regions, predominantly the left dlPFC and parts of the mPFC.

sFLU (*N* = 95), pFLU (*N* = 92), and TMT B/A (*N* = 64) performance on the other hand were not significantly associated with GMV at the given threshold.

For all significant associations (i.e. *p* < 0.005 FWE_TFCE_; RMET, H5PT, Stroop) overlapping associations with GMV were found within the left frontoinsular region (i.e. Broca triangular region; see Fig. 1) and within the bilateral PFC (i.e. dlPFC), at more liberal thresholds (i.e. i.e. *p* < 0.05 FWE_TFCE_) this pattern was more pronounced and included parts of the left basal ganglia. Explorative overlap analyses after applying this liberal threshold to all analyses (i.e. i.e. *p* < 0.05 FWE_TFCE_) highlighted similar regions with pronounced overlap within the left putamen, the left frontoinsular region and the bilateral dlPFC.

### Associations of Reading the Mind in the Eyes and executive functions test performance with cortical thickness

**Fig. 2:**
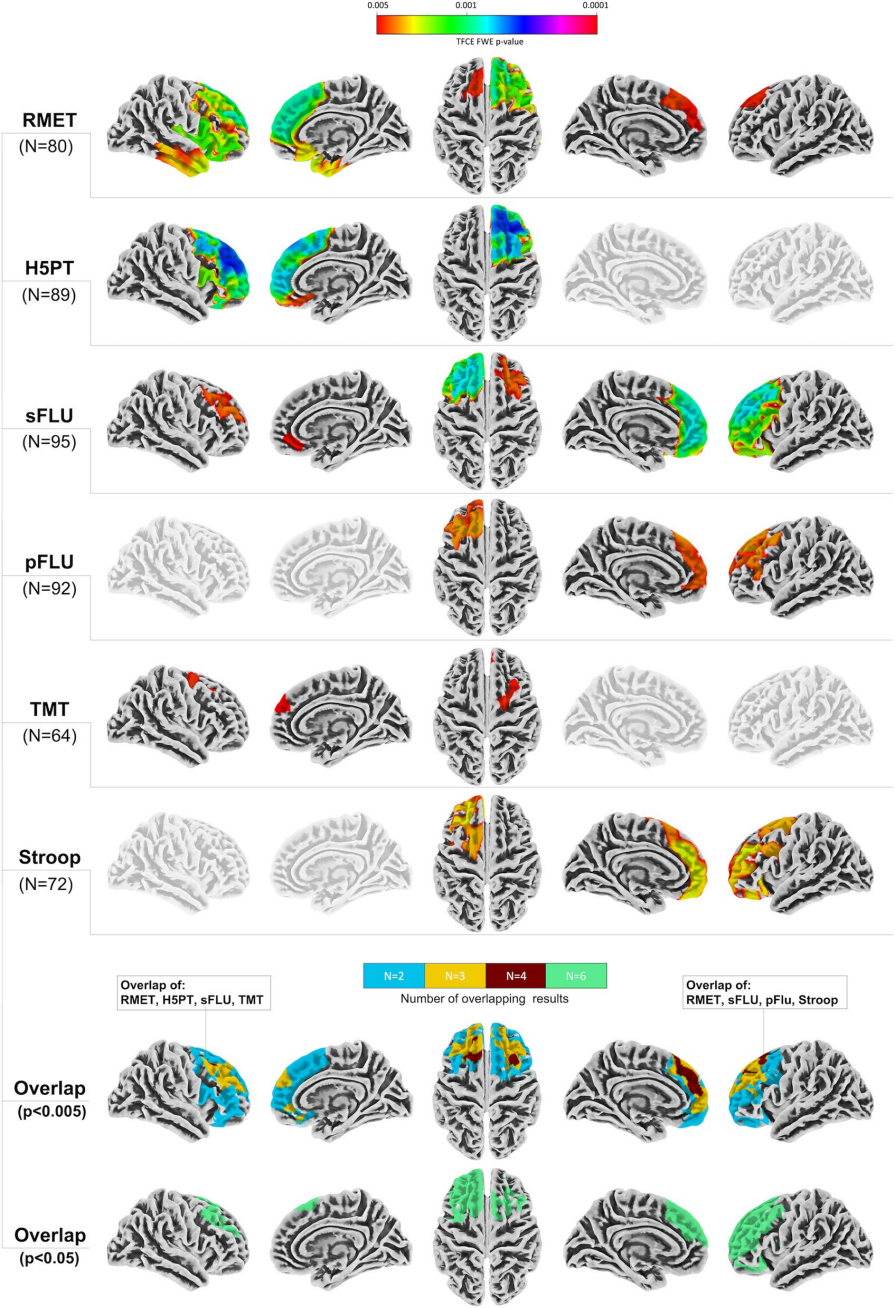
Associations of cortical thickness (CTH) with performance in the Reading the Mind in the Eyes Test (RMET) and five different tests reflective of executive function (EF). Results are thresholded at *p* < 0.005 FWE_TFCE_. All results indicate a positive relationship of test performance. The color bar illustrates the level of significance (i.e. pFWE_TFCE_). Bottom rows highlight overlapping regions of CTH results (please note that colors are not reflective of individual tests, but highlight the number of overlapping results only). The bottom row highlights the overlap of all six tests at a liberal threshold (*p*<0.05 FWE_TFCE_) for exploratory purposes. Left side of the brain is shown on the left (i.e. when both hemispheres are shown).* H5PT *Hamasch-Five-Point Test, *TMT *Trail Making Test B/A; *sFLU* semantic Fluency; *pFLU *phonemic Fluency

For detailed results see Fig. 2 and supplementary Table S3. Exclusively positive associations of CTH with better test performance were found for all analyses or, correspondingly, positive associations between atrophy and worse test performance (*p* < 0.005 FWE_TFCE_; the respective sample sizes are identical to the VBM analyses).

Multiple regression analyses showed – in parts widespread - positive associations with prefrontal CTH (mostly within mPFC and dlPFC) for test performance reflective of better EF (i.e. H5PT, sFLU, pFLU, TMT, Stroop) and ToM (i.e. RMET). While bilateral associations were found for RMET (right > left) and sFLU performance (left > right), H5PT and TMT were associated with right prefrontal CTH only. Stroop and pFLU associations were located within the left PFC. sFLU, RMET, Stroop and H5PT performance were additionally associated with orbitofrontal CTH. For RMET only, additional associations were found within the right frontoinsular region and insular cortex as well as polar, medial and lateral aspects of the right temporal lobe. Results overlapped within the right dlPFC (i.e. RMET, H5PT, sFLU, TMT) and the left mPFC and dlPFC (i.e. RMET, sFLU, pFLU, Stroop). Explorative overlap analyses after applying a liberal threshold to all analyses (i.e. i.e. *p* < 0.05 FWE_TFCE_) highlighted similar overlapping associations (i.e. bilateral mPFC and bilateral PFC, parts of the left OFC and frontoinsular region) for all six cognitive tests.

### Independent associations of Reading the Mind in the Eyes and executive functions test performance

As detailed above, both sFLU and H5PT were significantly associated with RMET performance within the GLM. Thus, we chose to investigate the respective associations of RMET after additional adjustment for both sFLU and H5PT (*N* = 77) and after individual adjustment for sFLU (*N* = 77) and H5PT (*N* = 80) respectively.

#### VBM

**Fig. 3:**
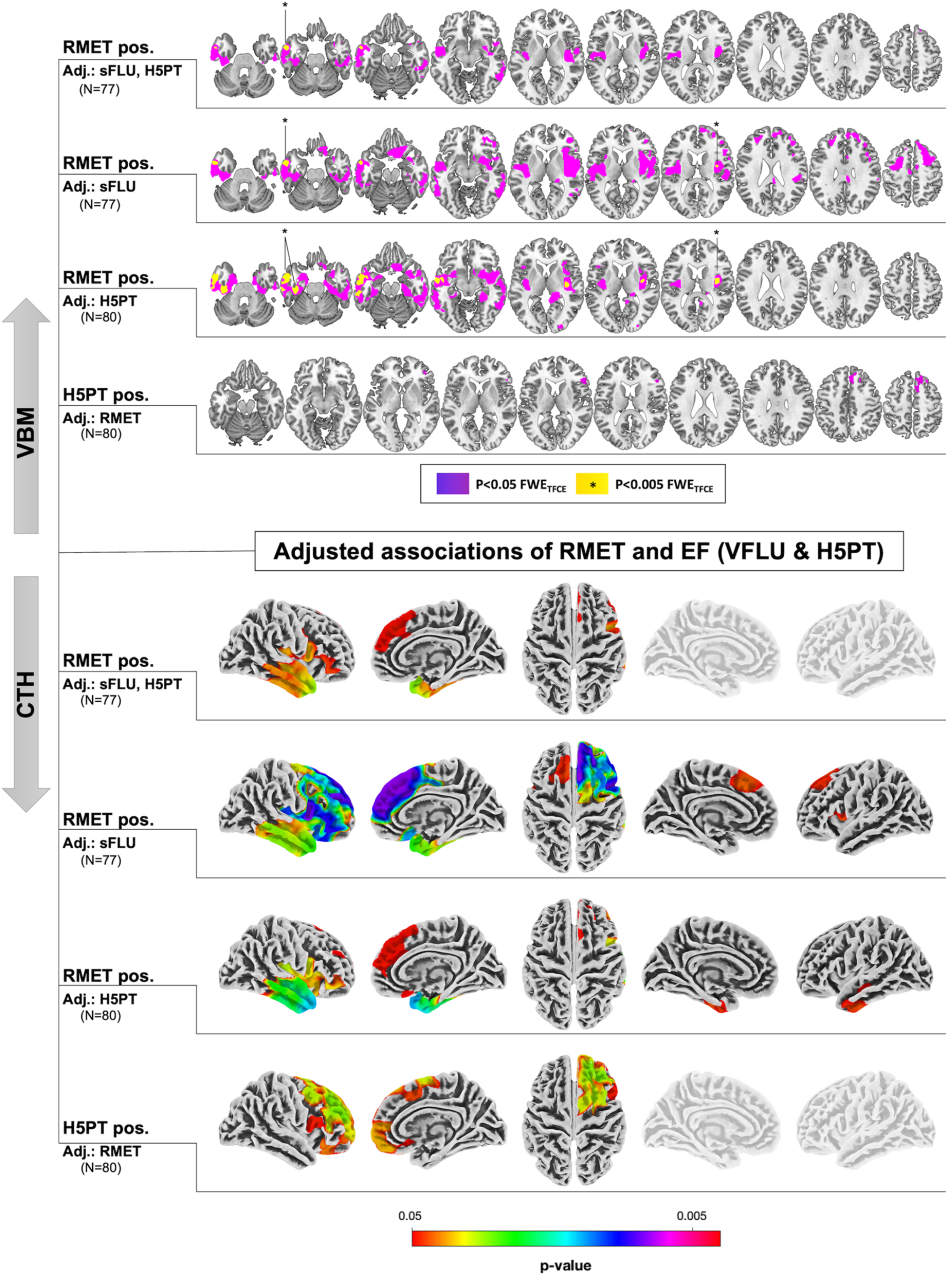
Associations of Reading the Mind in the Eyes (RMET) and executive function (EF) test performance with gray matter volume (GMV; top rows) and cortical thickness (CTH; bottom rows) after additional adjustment for the respective other test performance (i.e. RMET adjusted for sFLU and H5PT, combined and individually). Results are thresholded at *p* < 0.05 FWE_TFCE_ and *p* < 0.005 FWE_TFCE_ [voxel based morphometry (VBM) results for RMET only; indicated by warm yellow color]. Here, for better differentiation VBM results are illustrated with two separate monochromatic colormaps. All illustrated results indicate a positive relationship of test performance with GMV and CTH. For CTH results, the color bar illustrates the level of significance (i.e. pFWE_TFCE_). Left side of the brain is shown on the left (i.e. in sections and when both hemispheres are shown). *sFLU* semantic fluency; *H5PT *Hamasch-Five-Point Test

For detailed results please see Fig. 3 and supplementary Table S4. In all models (i.e. combined and individual additional adjustment for sFLU and H5PT performance, *p* < 0.005 FWE_TFCE_) we found GMV of the left temporal pole to be positively associated with better RMET performance. These associations were the strongest when adjusted for H5PT only. Here additional associations were found within the left medial temporal lobe (i.e. the parahippocampus) and the right insula at the given threshold (the latter was also evident after adjustment for sFLU only). Overlap of all three models was again observed within the left temporal pole. Liberal analyses (*p* < 0.05 FWE_TFCE_) showed more extensive positive associations within bilateral lateral and medial temporal cortical regions, the bilateral insula, and adjacent parts of the basal ganglia. Models adjusted for sFLU or H5PT alone, overall closely resembled the pattern observed in our primary analyses of RMET (i.e. models without adjustment for EF measures). At *p* < 0.005 FWE_TFCE_ no significant associations of better H5PT or sFLU performance with GMV were found in all models with adjustments for RMET. Here, liberal analyses at *p* < 0.05 FWE_TFCE_ showed positive associations of H5PT (but not sFLU) with GMV within the right PFC, including the right dlPFC and the right inferior frontal gyrus in the vicinity of the frontoinsular region with similar results in models adjusted for RMET alone and with additional adjustment for sFLU (the latter is not shown in Fig. 3). sFLU individually did not show any associations at a liberal *p* < 0.05 FWE_TFCE_ when corrected for RMET.

#### Cortical thickness

For detailed results please see Fig. 3 and supplementary Table S5. At *p* < 0.005 FWE_TFCE_ no significant associations of RMET and EF performance scores with CTH were found. More liberally analyzed (*p* < 0.05 FWE_TFCE_), better RMET performance scores – adjusted for sFLU and H5PT – were positively associated with CTH of the right anterior temporal lobe, most prominently the right temporal pole next to the insular cortex, adjacent frontal regions and the right medial PFC. After individual adjustment for sFLU a similar pattern of temporal, insular and prefrontal (right > left) associations emerged as compared to our primary analysis of RMET. Individual adjustment for H5PT again showed positive associations of RMET with bilateral temporal CTH (right > left), the right insula and adjacent prefrontal regions, whereas in the same model, H5PT showed extensive positive associations with right prefrontal CTH, including medial and dorsolateral aspects.

Models that included both sFLU and H5PT as well as models including sFLU only, did not show any significant associations for the respective EF measures after adjustments were made for RMET.

### Associations with structural covariance networks

Details on the individual components with respect to local maxima can be found as supplementary data (i.e. supplementary data SD1). Furthermore, we provide the results overview as provided via the GIFT Toolbox as supplement (i.e. supplementary data SD2), which includes more detailed graphical illustrations of the components, next to methodological aspects of the analysis.

**Fig. 4:**
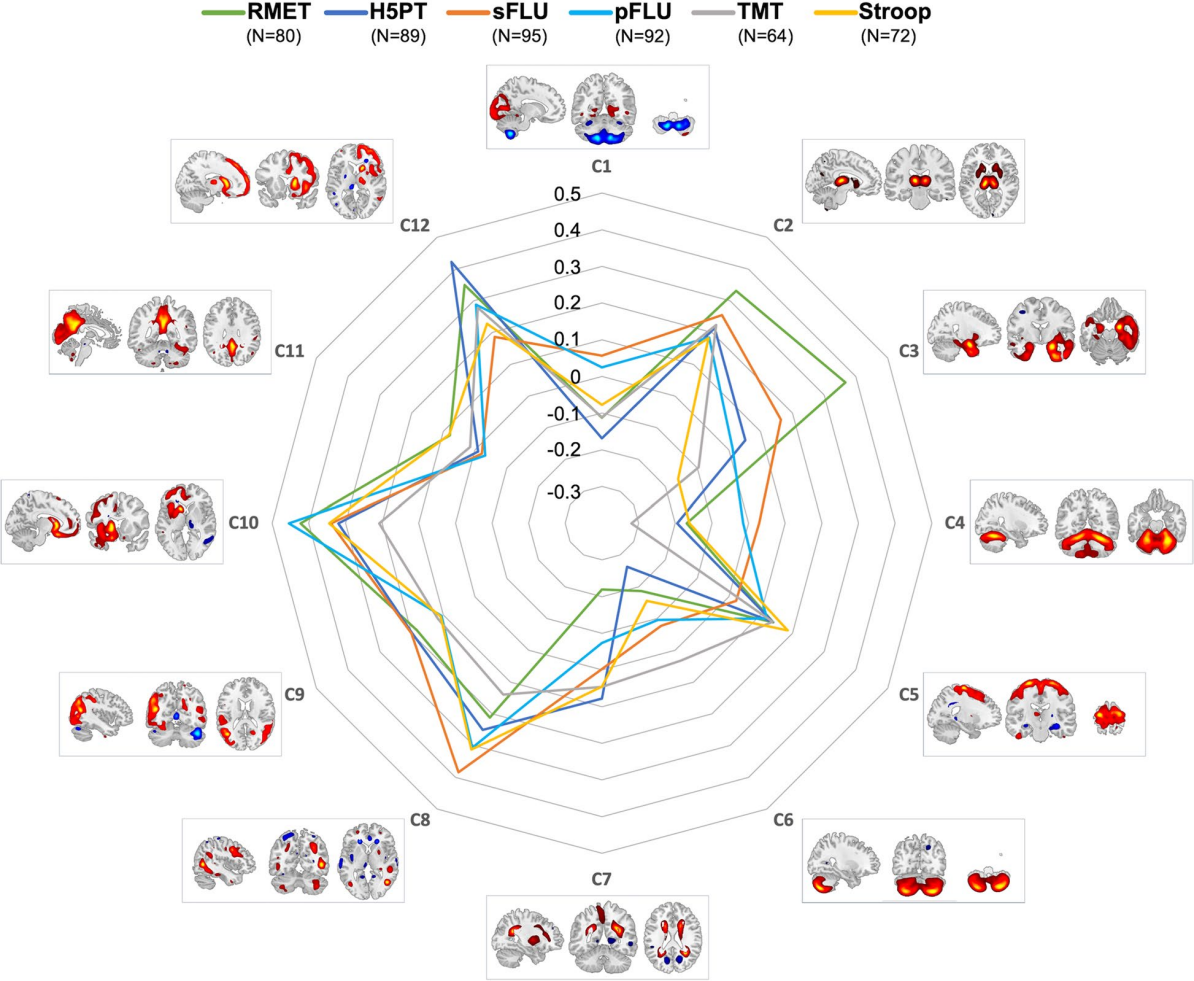
Radar chart illustrating the relationship of structural covariance networks (i.e. C1 – C12) with performance in the Reading the Mind in the Eyes Test (RMET) and executive function (EF) tests with the scale indicating spearman r correlation coefficients of partial correlation analyses adjusted for age and gender. Left side of the brain is shown on the left when both hemispheres are shown). *H5PT* Hamasch-Five-Point Test, *TMT *Trail Making Test B/A; *sFLU* semantic Fluency; *pFLU *phonemic Fluency. Please note that for the TMT lower values indicate better performance, therefore the correlation coefficients were inverted for illustrative purposes

Partial Spearman correlation analyses (corrected for age and gender) of the 12 independent components (C1 to C12; Fig. 4 and supplementary Tables S6) with RMET and EF measures yielded significant correlations (pFDR < 0.05) of RMET with the following independent components: C2 – a subcortical network mainly including the bilateral thalamus and parts of the striatum; C3 – a temporal network mainly characterized by medial and lateral temporal cortical regions including the temporal pole (right > left); C10 – a left frontoinsular network, mainly characterized by the left PFC including medial, orbitofrontal and dorsolateral aspects and the left insula, next to parts of the left caudate and left medial and lateral aspects of the temporal lobe; C12 a right frontoinsular network mainly characterized by the right PFC including medial, orbitofrontal and dorsolateral aspects and the right insula, next to parts of the right caudate.

Overall, tests reflective of EF showed partially overlapping correlations (pFDR < 0.05), which included C10 and C12 – but not C2 and C3 – and additionally C8, a structural network mainly characterized by the bilateral dlPFC but also parts of the bilateral parietotemporal cortex and the bilateral cerebellum.

### Comparisons with Neurosynth meta-analyses

**Fig. 5:**
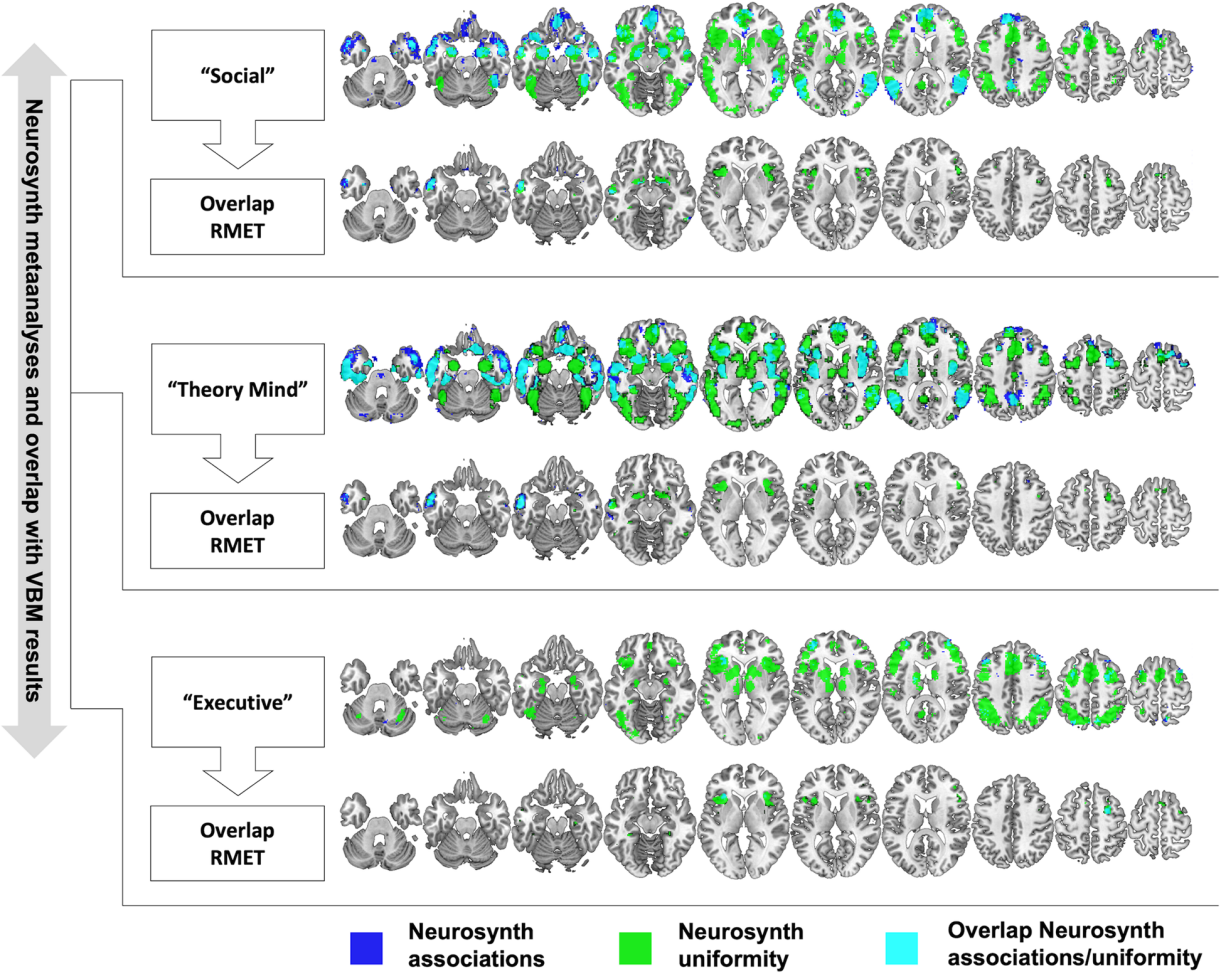
Term-based (i.e. “social”, “theory mind”, “executive”) Neurosynth meta-analyses with the corresponding overlap with voxel based morphometry (VBM) based brain structural correlates of the Reading the Mind in the Eyes Test (RMET) as determined in this study. Left side of the brain is shown on the left

Overall, Neurosynth term-based meta-analyses showed largely overlapping results of associations tests and uniformity tests for the respective term (see Fig. 5). Nevertheless, uniformity analyses tended to be more spatially extended.

Identical to Schroeter et al. [30] we found extensive activations for the term “social”, mainly within the mPFC, dlPFC, frontal operculum and the insular cortex, next to basal ganglia and thalamus, temporal cortical regions including the amygdala, the TPJ and the posterior cingulum.

Overlap with VBM correlates of RMET was observed within anterior parts of the left lateral temporal cortex and the bilateral frontal operculum and adjacent insular cortex as well as the bilateral putamen and the dlPFC. However, overlap of RMET with both associations test and uniformity test was mainly restricted to the anterior left temporal cortex. For the term “theory mind” Neurosynth meta-analyses yielded a very similar pattern of activations and overlap with VBM RMET results.

For the term “executive”, Neurosynth showed a partially overlapping pattern of activations with respect to the other terms, however, activations within prefrontal (except for the OFC) and parietal regions were more extent. Overlap with RMET was found within the bilateral insula and the dlPFC (mostly right) whereas the temporal cortex only showed discrete overlap.

Conversely, location based meta-analyses using the peak voxel location from our adjusted VBM analyses of RMET (i.e. after adjustement for both sFLU and H5PT and sFLU or H5PT alone: left temporal pole MNI_xyz_: -57 3–22 mm, corresponding to rounded Neurosynth coordinates − 56 4–22 mm) yielded the term “mind tom” (z-score = 5.55; posterior probability = 0.88; functional connectivity (r) = 0.38; Meta-analytic coactivation (r) = 0.35) as sixth strongest meta-analytic association out of 1334 entries, next to “theory mind” as seventh strongest association.

## Discussion

In this large sample of bvFTD patients, we investigated the brain structural correlates of mentalizing capabilities (i.e. ToM, a key aspect of SC) via a modified RMET and five different tests reflective of EF. Here we find better RMET performance to be positively associated with GMV within temporal cortical, insular and prefrontal brain regions. Analyses of CTH showed a similar pattern, however more pronounced for the right hemisphere. Associations of EF task performance, on the other hand, were mostly limited to the PFC including the mPFC and the dlPFC, as well as the insular cortex. Post-hoc analyses showed independent associations of RMET performance (i.e. after adjustment for EF performance) with GMV and CTH mainly within the bilateral temporal lobes (most prominently within the left temporal pole) and the bilateral insula. Independent associations of EF were only observed for H5PT and were largely limited to the PFC. For RMET and all tests of EF, we found GMV associations to overlap within the left insula and parts of the dlPFC, exploratory analyses at more liberal thresholds yielded additional overlapping regions, most prominently the left putamen. Source-based morphometry analyses of structural covariance networks yielded comparable results, with RMET performance being more strongly associated with temporal networks and EF performance being more strongly associated with prefrontal networks. Making use of the Neurosynth database, overlap of RMET with functional networks commonly associated with both SC and EF was again primarily located within the temporal and insular region and the dlPFC. Additionally, location based meta-analyses (also within the Neurosynth database) of independent RMET associations (i.e. after adjusting for EF) within the left temporal pole were found to be very strongly associated with terms related to SC and TOM in particular (i.e. “mind tom” “theory mind”) indicating a critical role of anterior temporal lobe atrophy in the development of social cognitive deficits in bvFTD.

Summarized these results indicate that bvFTD-related deficits of ToM and EF may be attributable to atrophy of distinct brain regions, with ToM and EF deficits being more related to temporal and prefrontal atrophy, respectively. Interestingly, deficits of ToM and EF appear to share the insula and distinct regions within the PFC (i.e. dlPFC) as common neural correlates.

Several previous neuroimaging studies have investigated brain structural correlates of SC in bvFTD. Although test batteries and corresponding aspects of SC (e.g. ToM, emotion processing, moral reasoning) differed across these studies, the overall pattern of associated brain regions is comparable to our results, pointing to a discrete network of medial, prefrontal, orbitofrontal, insular and anterior temporal cortical regions being involved in bvFTD related SC deficits, including ToM [8, 18–26]. Since bvFTD is a rare disease previous studies were done with comparably small sample sizes (i.e. all *N* < 30). For healthy humans on the other hand, an extensive number of neuroimaging studies have investigated the neurobiological underpinnings of SC and TOM, with meta-analyses across fMRI studies – including our Neurosynth analysis – corroboratively pointing to a network that includes part of the PFC, the anterior cingulate cortex, the TPJ and the temporal lobes [15–17]. In our study, RMET correlates were predominantly located within the temporal cortex – including both lateral and medial aspects and the temporal pole – a pattern that remained evident even after controlling for measures of EF. Note that our study represents a lesion approach, herewith validating results of functional studies in healthy subjects.

Important functions of the lateral temporal lobe include object and face recognition together with language and conceptual processing, all processes relevant in everyday social interactions. The medial temporal lobe with its prominent role in mnestic functions is implicated in memory retrieval of socially relevant information and experiences, while anterior parts of the temporal lobe and the temporal pole are thought to provide entire social semantic concepts [50, 51]. On the other hand, the amygdala, located within the anterior temporal lobe, has a prominent role in emotional processing, with human neuroimaging studies showing consistent activation in response to different kinds of facial expressions [50, 52–55].

We also observed positive associations of RMET within the bilateral dlPFC, however, these findings were strongly diminished after adjustments were made for EF. Nevertheless, considerable overlap of our results with functional neuroimaging studies was found within the bilateral dlPFC, when compared to Neurosynth based meta-analyses of SC and TOM. The dlPFC – as most parts of the PFC – is critically involved in higher cognitive processes, most prominently EF [56, 57]. Human behavior in its complexity depends on EF due to the necessity of constant evaluation, reevaluation, self-control and flexible adjustment, a concept that naturally also applies to complex human interactions [14, 58]. With respect to the dlPFC, growing evidence from both human and animal research indicates a prominent role for this region in tracking and cognitive evaluation of social interactions, its outcomes and subsequent adaptations [59–61] with cognitive perspective-taking, an aspect of SC closely related to ToM, being critically dependent on dlPFC function [62].

In our study, worse performance of EF tests was mainly related to prefrontal atrophy, including ventromedial, dorsomedial and dorsolateral aspects of the PFC. Although results differed to some degree between individual tests, the main pattern is in accordance with fMRI studies in healthy humans and studies in dementia based on glucose metabolism. Here, meta-analyses found EF to be dependent on a distinct network of prefrontal regions [3, 63], also highly converging with results from our Neurosynth meta-analysis. Nevertheless, the meta-analysis by Niendam et al. also highlighted the parietooccipital cortex, the insula and the basal ganglia [63], brain regions that only partially were related to EF in our sample. Of note, the relationship between brain structure and cognitive functions is complex and results of healthy participants may not be entirely comparable to diseased participants with cognitive deficits due to profound pathological brain changes, as in our sample of patients with bvFTD.

Overlap between structural correlates of RMET and EF was consistently observed for the anterior insular cortex and adjacent frontal regions including parts of the inferior frontal cortex, as well as distinct regions within the PFC, partially corresponding to the dlPFC. As detailed above, a role for the dlPFC in both SC and EF is well established. The insula on the other hand serves a number of roles with processing internal signals regarding the physiological state of the body (i.e. interoception) being one of its main functions. Due to a large body of evidence, it has been postulated that the central representation of bodily states (e.g. hunger, thirst, pain) within the insula may be the neurobiological foundation of emotions and its awareness [64–66].

While the posterior insula receives as primary homeostatic cortex directly information on bodily states, the anterior insula is supposed to be primarily involved in its emotional processing. Interestingly, neuroimaging studies showed identical anterior insular activation patterns when directly experiencing bodily and emotional distress (e.g. disgust and pain) as compared to seeing facial expressions of other people making these experiences. Based on these observations, the insular cortex has been proposed to process one’s own emotional state but also to predict the emotional states of others [67]. Of note, large clusters of von Economo neurons (i.e. “Mirror Neurons”) can be found in high density within the frontoinsular cortex, a special population of neurons which is thought to be critically involved in socioemotional functions and vicarious learning (i.e. via observing others), a crucial aspect of human social behavior [67–71]. Remarkably, bvFTD seems to affect von Economo neurons in particular [2].

Based on extensive evidence from human neuroimaging studies and its connectivity profile, the insula, and its anterior aspects in particular, has been conceptualized as a hub for multimodal information with an overarching function as switch gate for a number of different brain networks such as salience, default mode and executive networks [72] thus enabling the integration of emotional and cognitive information. With this perspective, our findings of consistent overlap for ToM and EF deficits within the insula seem plausible within a simplified framework of the insula functioning as common linking structure between social cognitive processing on the level of perceptive, and affective processing – primarily located within the temporal lobe – and its cognitive evaluation and integration in behavioral patterns, which in turn largely depends on the prefrontal cortex, including the dlPFC.

Finally, we want to place our results into a conceptual framework of bvFTD and its symptoms as recently developed with the MARS approach [2, 29, 30]. Our results underline the importance of atrophy in frontolateral and frontomedian structures for deficits in EF in bvFTD. On the other hand, results support the importance of atrophy in anterior insula, basal ganglia and frontal structures for deficits in SC and emotion processing, but indicate also important contributions of the temporal lobes to those deficits. Moreover, our results support the hypothesis that deficits in EF and SC in bvFTD share associated and involve dissociated brain structures at the same time.

However, several limitations need to be considered, thus our findings should be kept in perspective. First, we did include patients with possible bvFTD, next to patients with both probable and definite bvFTD. Although all patients with possible bvFTD fulfilled clinical diagnostic criteria of bvFTD and were diagnosed after multidisciplinary discussion of each case, we cannot entirely rule out the possibility of bvFTD phenocopies in our sample, such as primary psychiatric disorder or frontal variant Alzheimer’s disease (AD). With respect to the latter, only limited data regarding CSF based AD pathology were available. However, these data did not indicate a systematic bias (data not shown), while comparable frequencies of co-pathology could be observed as previously published [73]. Moreover, longitudinal stability of (possible and probable) bvFTD diagnosis in our cohort prevent a systematic bias of phenocopy cases. Next, the modified RMET as used in this study only captures one aspect of SC (i.e. ToM). In this context, it also has to be noted that other tests reflective of ToM (e.g. Faux Pas Test, Sarcasm Detection) may provide more accurate information with respect to mentalizing deficits in bvFTD [74]. This is a multi-centric study with neuroimaging data having been acquired on different scanners. Although imaging protocols have been harmonized across participating centers via standard operating procedures, scanner effects can still affect results. Due to aforementioned reasons we did choose not to control for scanner site, in order to prevent excessive loss of information, and assuming high statistical power and validity due to a very large cohort. Of note, our analyses involved mainly correlation analyses, where different scanners and protocols are not assumed to systematically bias results. Data were not equally available for all cognitive tests, resulting in differences with respect to the number of subjects analyzed for each test, which may have had an impact on our results and the level of significance. Since bvFTD is a rare disease and the acquisition of good quality data is cumbersome, we chose not to exclude data for this particular reason. Next, some discrepancies can be noted, when comparing results of VBM and CTH analyses, although the directionalities and spatial patterns (i.e. temporoinsular pronunciation for RMET analyses and prefrontal pronunciation for EF analyses) were similar for both analyses. VBM captures a number of different brain structural aspects including CTH, whereas analyses of CTH are exclusive of other structural variables, thus highlighting CTH only. While both modalities provide information regarding structural aspects of the brain, they should be considered complimentary but not equivalent [75, 76].

## Conclusion

In this sample of bvFTD patients we find dissociating atrophy patterns associated with ToM and EF deficits. Here, ToM deficits were more related to atrophy of the lateral and medial temporal lobes including the temporal pole, brain regions implicated in emotional processing and processing of socially relevant information such as facial recognition and social semantic concepts. Executive function deficits, on the other hand, were more related to atrophy of prefrontal regions in line with current concepts of EF’s neurobiological underpinnings. Common overlapping atrophy patterns were identified within the insular cortex and the dlPFC. The insula serves a number of functions and has been proposed as a common integrating hub for a number of cognitive processes and networks, whereas the dlPFC is critically involved in cognitive control – a partial aspect of EF – which in turn is required for complex social behaviors.

## Supplementary Information

Supplementary Material 1.

Supplementary Material 2.

Supplementary Material 3.

## Data Availability

Data are available from the corresponding author on reasonable request
