# Supplementary material for: Dissecting neural correlates of theory of mind and executive functions in behavioral variant frontotemporal dementia
Source: Alzheimers Res Ther. 2024 Oct 26;16:237. doi: 10.1186/s13195-024-01596-4 (PMC11515257; doi:10.1186/s13195-024-01596-4)
Supplement: Supplementary file 2 — Supplementary Material 2. [file 13195_2024_1596_MOESM2_ESM.docx]

**Supplementary Tables**

The following tables provide additional information for VBM (i.e. gray matter volume) and cortical thickness associations with Reading the Mind in the Eyes and executive functions test performance (i.e. Tables S2 – S5). Results of correlational analyses of SBM derived gray matter structural covariance networks with Reading the Mind in the Eyes and executive functions test performance are highlighted in Table S6. For additional information and references we refer to the main file.

| Table S2: Associations of gray matter volume with Reading the Mind in the Eyes and executive functions test performance | | | | |
| --- | --- | --- | --- | --- |
| Brain Region (Brodman Area) | equivk | p(FWE-corr) | TFCE | x,y,z {mm} |
| Positive associations of GMV with RMET (N=80) | | | | |
| Left-Temporalpole (38), Middle Temporal Gyrus | **11330** | **0.000** | **3319.98** | **-56 2 -24** |
| Left-SupTempGyrus (22) |  | 0.000 | 3166.20 | -58 -4 -14 |
| Left-InfTempGyrus (20) |  | 0.000 | 3153.78 | -58 -8 -34 |
| Right Insula (13) | **11431** | **0.001** | **2953.13** | **34 -4 15** |
| Right-Insula (13) |  | 0.001 | 2693.25 | 39 -14 3 |
| Right-MedTempGyrus (21), |  | 0.001 | 2680.82 | 62 -27 -15 |
| Right-FrontEyeFields (8), Middle Frontal Gyrus | **1943** | **0.003** | **2321.92** | **27 24 44** |
| Right-dlPFC(dorsal) (9), Superior Frontal Gyrus |  | 0.003 | 2299.57 | 30 33 34 |
| Right-Medial Frontal Gyrus (6) |  | 0.003 | 2295.51 | 21 0 54 |
| Right-Lentiform Nucleus, Putamen | **10** | **0.003** | **2320.12** | **33 -15 -9** |
| Right-dlPFC(lat) (46), Inferior Frontal Gyrus | **188** | **0.004** | **2203.40** | **42 42 3** |
| Left-FrontEyeFields (8), Middle Frontal Gyrus | **206** | **0.004** | **2155.70** | **-22 22 40** |
| Left-dlPFC(dorsal) (9), Medial Frontal Gyrus |  | 0.004 | 2141.07 | -22 34 34 |
| Left-dlPFC(dorsal) (9), Medial Frontal Gyrus | **1** | **0.005** | **2104.24** | **-21 38 28** |
| Positive associations of GMV with H5PT (N=89) | | | | |
| Right-FrontEyeFields (8), Superior Frontal Gyrus | **11764** | **0.000** | **2996.32** | **18 28 50** |
| Right-FrontEyeFields (8), Superior Frontal Gyrus |  | 0.000 | 2945.03 | 26 26 54 |
| Right-FrontEyeFields (8), Middle Frontal Gyrus |  | 0.000 | 2919.77 | 22 30 40 |
| Left-Insula (13) | **2584** | **0.001** | **2498.14** | **-32 -8 12** |
| Left-Claustrum |  | 0.001 | 2482.86 | -30 4 10 |
| Left-Broca-Operc (44) |  | 0.001 | 2454.97 | -42 9 9 |
| Left-PreMot+SuppMot (6), Middle Frontal Gyrus | **2188** | **0.002** | **2366.04** | **-26 -2 51** |
| Left-Middle Frontal Gyrus (6) |  | 0.002 | 2337.46 | -26 4 58 |
| Left-FrontEyeFields (8), Precentral Gyrus |  | 0.003 | 2101.85 | -40 15 36 |
| Left-AntPFC (10), Middle Frontal Gyrus | **1207** | **0.002** | **2201.18** | **-33 50 15** |
| Left-dlPFC(lat) (46), Inferior Frontal Gyrus |  | 0.003 | 2092.34 | -44 38 9 |
| Left-dlPFC(lat) (46), Inferior Frontal Gyrus |  | 0.003 | 2079.08 | -44 46 4 |
| Left-DorsalACC (32) | **61** | **0.005** | **1969.13** | **-10 46 2** |
| Left-AntPFC (10), Medial Frontal Gyrus | **44** | **0.005** | **1951.10** | **-9 62 3** |
| Left-AntPFC (10), Medial Frontal Gyrus | **8** | **0.005** | **1943.62** | **-18 56 -8** |
| Positive associations of GMV with Stroop (N=72) | | | | |
| Left-Superior Frontal Gyrus | **2415** | **0.002** | **2609.65** | **-12 51 26** |
| Left-AntPFC (10) |  | 0.002 | 2593.32 | -24 51 2 |
| Left-dlPFC(dorsal) (9), Medial Frontal Gyrus |  | 0.002 | 2540.52 | -22 38 26 |
| Left-FrontEyeFields (8) | **599** | **0.003** | **2383.48** | **-18 26 44** |
| Left-FrontEyeFields (8), Superior Frontal Gyrus |  | 0.003 | 2368.94 | -24 24 50 |
| Left-FrontEyeFields (8), Middle Frontal Gyrus |  | 0.003 | 2346.01 | -28 20 42 |
| Left-ParsOrbitalis (47) | **437** | **0.003** | **2344.83** | **-28 24 -15** |
| Left-dlPFC(dorsal) (9), Middle Frontal Gyrus | **294** | **0.003** | **2319.94** | **-42 20 32** |
| Left-Broca-Operc (44), Inferior Frontal Gyrus |  | 0.004 | 2268.79 | -39 10 22 |
| Left-Insula (13) | **104** | **0.005** | **2183.42** | **-36 14 3** |
| Left-Broca-Triang (45), Inferior Frontal Gyrus | **24** | **0.005** | **2176.54** | **-42 21 10** |
| Results are listed at p < 0.005 FWE_TFCE_ whole-brain corrected with corresponding cluster size equivalent (equivk), TFCE scores and MNI coordinates. Bold data indicate primary peaks and non-bold data indicate secondary peaks within a cluster. BA, [Brodmann area](https://www.sciencedirect.com/topics/neuroscience/brodmann-area); *RMET* Reading the Mind in the Eyes Test; *H5PT* Hamasch-Five- Point Test; | | | | |

| Table S3: Associations of cortical thickness with Reading the Mind in the Eyes and executive functions test performance | | | | |
| --- | --- | --- | --- | --- |
| Brain Region (Brodman Area) | **equivk** | **p(FWE-corr)** | **TFCE** | **x,y,z {mm}** |
| Positive associations of CTH with RMET (N=80) | | | | |
| Right-dlPFC(lat) (46), Inferior Frontal Gyrus | 7104 | 0.002 | 83492.47 | 43 36 6 |
| Right-Insula (13) |  | 0.002 | 81656.58 | 35 -21 8 |
| Right-OrbFrontal (11), Rectal Gyrus |  | 0.002 | 75418.23 | 4 25 -22 |
| Right-Parahipp (36) | **1959** | **0.003** | **75252.08** | **24 -8 -29** |
| Left-FrontEyeFields (8), Middle Frontal Gyrus | **847** | **0.004** | **65360.27** | **-24 22 36** |
| Positive associations of CTH with H5PT (N=89) | | | | |
| Right-dlPFC(dorsal) (9), Middle Frontal Gyrus | **5826** | **0.000** | **100170.88** | **27 36 25** |
| Right-FrontEyeFields (8), Middle Frontal Gyrus |  | 0.000 | 96744.09 | 27 12 46 |
| Right-ParsOrbitalis (47), Inferior Frontal Gyrus |  | 0.001 | 85934.09 | 25 34 -11 |
| Positive associations of CTH with sFLU (N=95) | | | | |
| Left-AntPFC (10), Superior Frontal Gyrus | **5628** | **0.001** | **91289.10** | **-23 45 25** |
| Left-dlPFC(dorsal) (9), Medial Frontal Gyrus |  | 0.001 | 90282.26 | -6 46 23 |
| Left-FrontEyeFields (8), Superior Frontal Gyrus |  | 0.001 | 89569.68 | -14 27 51 |
| Right-dlPFC(dorsal) (9), Middle Frontal Gyrus | **1162** | **0.003** | **65006.12** | **28 37 28** |
| Right-dlPFC(dorsal) (9), Superior Frontal Gyrus |  | 0.004 | 64023.06 | 44 34 30 |
| Right-FrontEyeFields (8), Middle Frontal Gyrus |  | 0.004 | 63509.69 | 23 29 43 |
| Right-OrbFrontal (11), Anterior Cingulate | **185** | **0.005** | **60426.97** | **10 38 -9** |
| Positive associations of CTH with pFLU (N=92) | | | | |
| Left-FrontEyeFields (8), Superior Frontal Gyrus | **2094** | **0.003** | **69590.65** | **-12 30 52** |
| Left-dlPFC(dorsal) (9), Middle Frontal Gyrus |  | 0.003 | 69114.23 | -35 31 35 |
| Left-dlPFC(dorsal) (9), Medial Frontal Gyrus |  | 0.004 | 65741.08 | -7 46 21 |
| Positive associations of CTH with Stroop (congruent/incongruent; N=72) | | | | |
| Left-dlPFC(dorsal) (9), Superior Frontal Gyrus | 4031 | 0.002 | 77919.41 | -24 42 29 |
| Left-AntPFC (10), Superior Frontal Gyrus |  | 0.003 | 75158.31 | -23 55 5 |
| Inverse associations of CTH with TMT B/A ratio (N=64) |  |  |  |  |
| Right-PreMot+SuppMot (6), Middle Frontal Gyrus | **483** | **0.004** | **79361.80** | **23 2 52** |
| Right-FrontEyeFields (8), Middle Frontal Gyrus |  | 0.005 | 77503.12 | 34 21 49 |
| Right-dlPFC(dorsal) (9), Medial Frontal Gyrus | **81** | **0.005** | **75840.11** | **7 53 26** |
| Results are listed at p < 0.005 FWE_TFCE_ whole-brain corrected with corresponding cluster size equivalent (equivk), TFCE scores and MNI coordinates. Bold data indicate primary peaks and non-bold data indicate secondary peaks within a cluster. BA, Brodmann area. *RMET* Reading the Mind in the Eyes Test; *H5PT* Hamasch-Five- Point Test; *sFLU* semantic Fluency; *pFLU* phonemic Fluency; *TMT* Trail Making Test; | | | | |

| Table S4: Associations of gray matter volume with mutually adjusted Reading the Mind in the Eyes and executive functions test performance | | | | |
| --- | --- | --- | --- | --- |
| Brain Region (Brodman Area) | equivk | p(FWE-corr) | TFCE | x,y,z {mm} |
| Positive associations of GMV with RMET, adjusted for sFLU&H5PT(N=77) | | | | |
| Left-Temporalpole (38), Middle Temporal Gyrus | **4672** | **0.002** | **2207.73** | **-57 3 -22** |
| Left-SupTempGyrus (22) |  | 0.005 | 1941.57 | -58 -2 -14 |
| Left-SupTempGyrus (22) |  | 0.010 | 1732.51 | -50 0 -14 |
| Right-Insula (13) | **6204** | **0.011** | **1682.45** | **36 -4 18** |
| Right-Insula (13) |  | 0.014 | 1607.98 | 40 -14 2 |
| Right-Insula (13) |  | 0.017 | 1528.64 | 36 -14 22 |
| Left-Claustrum | **1278** | **0.027** | **1371.24** | **-34 -12 10** |
| Left-PrimSensory (1), Postcentral Gyrus |  | 0.036 | 1274.01 | -54 -15 14 |
| Left-PrimSensory (1) |  | 0.041 | 1228.21 | -46 -14 18 |
| Right-Lentiform Nucleus, Putamen | **6** | **0.028** | **1364.89** | **33 -15 -9** |
| Right-Parahipp (36) | **2** | **0.046** | **1186.34** | **34 -30 -14** |
| Right-OrbFrontal (11), Rectal Gyrus | **41** | **0.046** | **1186.30** | **6 20 -24** |
| Right-Temporalpole (38), Superior Temporal Gyrus | **65** | **0.047** | **1179.09** | **36 8 -24** |
| Left-Parahipp (36), Uncus | **7** | **0.050** | **1160.87** | **-16 0 -34** |
| Positive associations of GMV with RMET, adjusted for sFLU ( N=77) | | | | |
| Left-Temporalpole (38), Middle Temporal Gyrus | **9940** | **0.002** | **2363.80** | **-57 3 -22** |
| Left-SupTempGyrus (22) |  | 0.005 | 2058.96 | -58 -2 -14 |
| Left-Insula (13) |  | 0.006 | 1948.20 | -32 -9 15 |
| Right-Insula (13) | **23618** | **0.005** | **2049.56** | **34 -4 16** |
| Right-Fusiform (37) |  | 0.007 | 1915.06 | 57 -54 -9 |
| Right-Fusiform (37) |  | 0.007 | 1903.27 | 60 -46 -9 |
| Right-ParsOrbitalis (47), Inferior Frontal Gyrus | 1 | 0.012 | 1705.59 | 30 34 -8 |
| Right-Lentiform Nucleus, Putamen | 7 | 0.022 | 1493.62 | 33 -15 -9 |
| Left-PreMot+SuppMot (6) | **3391** | **0.025** | **1439.23** | **-24 -2 58** |
| Left-PreMot+SuppMot (6), Middle Frontal Gyrus |  | 0.030 | 1374.27 | -33 -8 54 |
| Left-PreMot+SuppMot (6), Middle Frontal Gyrus |  | 0.030 | 1372.47 | -26 -8 64 |
| Right-Thalamus | **197** | **0.041** | **1261.36** | **22 -30 4** |
| Right-Cingulate Gyrus (23) | **170** | **0.045** | **1226.15** | **4 -30 27** |
| Left-InfTempGyrus (20) | **1** | **0.046** | **1218.49** | **-50 -27 -22** |
| Left-PreMot+SuppMot (6), Medial Frontal Gyrus | **109** | **0.046** | **1215.86** | **-6 -18 54** |
| Right-VentAntCing (24) | **328** | **0.046** | **1214.15** | **9 0 36** |
| Right-FrontEyeFields (8) |  | 0.047 | 1210.38 | 3 14 38 |
| Left-DorsalACC (32) |  | 0.047 | 1206.93 | -6 14 36 |
| Right-dlPFC(dorsal) (9), Middle Frontal Gyrus | **126** | **0.047** | **1206.86** | **40 16 28** |
| Right-Broca-Operc (44), Inferior Frontal Gyrus |  | 0.050 | 1188.67 | 46 9 30 |
| Left-FrontEyeFields (8), Medial Frontal Gyrus | **59** | **0.048** | **1203.82** | **-12 27 51** |
| Left-FrontEyeFields (8), Superior Frontal Gyrus |  | 0.048 | 1196.86 | -12 28 60 |
| Left-FrontEyeFields (8), Superior Frontal Gyrus |  | 0.049 | 1194.80 | -10 20 54 |
| Left-PreMot+SuppMot (6), Medial Frontal Gyrus | **1** | **0.048** | **1203.65** | **-9 15 54** |
| Left-PreMot+SuppMot (6), Superior Frontal Gyrus | **4** | **0.049** | **1195.14** | **-16 21 52** |
| Positive associations of GMV with RMET, adjusted for H5PT (N=80) | | | | |
| Left-Temporalpole (38), Middle Temporal Gyrus | **12159** | **0.001** | **2510.27** | **-56 3 -22** |
| Left-SupTempGyrus (22) |  | 0.001 | 2390.81 | -57 -2 -15 |
| Left-MedTempGyrus (21) |  | 0.002 | 2287.44 | -56 -8 -22 |
| Right-PrimMotor (4) | **15326** | **0.003** | **2084.39** | **36 -6 16** |
| Right-Insula (13) |  | 0.004 | 2009.49 | 40 -12 0 |
| Right-Claustrum |  | 0.004 | 1972.24 | 36 -18 -3 |
| Right-Lentiform Nucleus, Putamen | **12** | **0.007** | **1775.06** | **33 -15 -9** |
| Right-SecVisual (18), Middle Occipital Gyrus | **135** | **0.043** | **1182.20** | **27 -92 4** |
| Right-SecVisual (18), Middle Occipital Gyrus |  | 0.046 | 1157.76 | 32 -86 8 |
| Left-Broca-Triang (45), Insula | **154** | **0.047** | **1147.92** | **-42 20 8** |
| Left-Broca-Triang (45), Inferior Frontal Gyrus |  | 0.049 | 1132.94 | -36 26 9 |
| Left-Broca-Operc (44), Insula | **6** | **0.050** | **1129.73** | **-32 14 14** |
| Positive associations of GMV with H5PT, adjusted for RMET (N=80) | | | | |
| Right-FrontEyeFields (8), Superior Frontal Gyrus | **1446** | **0.023** | **1365.49** | **16 26 51** |
| Right-FrontEyeFields (8), Superior Frontal Gyrus |  | 0.025 | 1346.25 | 16 34 52 |
| Right-FrontEyeFields (8), Medial Frontal Gyrus |  | 0.028 | 1306.46 | 9 32 46 |
| Right-Broca-Triang (45), Inferior Frontal Gyrus | **268** | **0.034** | **1240.59** | **50 27 10** |
| Right-Broca-Operc (44), Inferior Frontal Gyrus |  | 0.042 | 1177.00 | 57 21 8 |
| Right-dlPFC(lat) (46), Inferior Frontal Gyrus | **91** | **0.036** | **1226.95** | **40 42 0** |
| Right-dlPFC(dorsal) (9), Middle Frontal Gyrus | **11** | **0.050** | **1125.63** | **40 32 21** |
| Results are listed at p < 0.05 FWE_TFCE_ whole-brain corrected with corresponding cluster size equivalent (equivk),  TFCE scores and MNI coordinates. Bold data indicate primary peaks and non-bold data indicate secondary peaks  Within a cluster. BA, [Brodmann area](https://www.sciencedirect.com/topics/neuroscience/brodmann-area). *RMET* Reading the Mind in the Eyes Test; *H5PT* Hamasch-Five- Point Test; *sFLU* semantic Fluency; | | | | |

| Table S5: Associations of cortical thickness with mutually adjusted Reading the Mind in the Eyes and executive functions test performance | | | | |
| --- | --- | --- | --- | --- |
| Brain Region (Brodman Area) | equivk | p(FWE-corr) | TFCE | x,y,z {mm} |
| Positive associations of CTH with RMET, adjusted for sFLU&H5PT (N=77) | | | | |
| Right-Insula (13) | **1748** | **0.045** | **28336.53** | **35 6 7** |
| Right-AntPFC (10), Medial Frontal Gyrus | **472** | **0.048** | **27469.87** | **7 53 19** |
| Positive associations of CTH with RMET, adjusted for sFLU (N=77) | | | | |
| Right-FrontEyeFields (8), Medial Frontal Gyrus | **11065** | **0.007** | **57724.39** | **7 35 40** |
| Right-AntPFC (10), Medial Frontal Gyrus |  | 0.007 | 57225.00 | 7 53 19 |
| Right-FrontEyeFields (8), Cingulate Gyrus |  | 0.007 | 56929.86 | 9 20 32 |
| Left-FrontEyeFields (8), Medial Frontal Gyrus | **807** | **0.041** | **29917.00** | **-5 25 44** |
| Left-Broca-Operc (44), Insula | **582** | **0.042** | **29696.02** | **-42 5 8** |
| Positive associations of CTH with RMET, adjusted for H5PT(N=80) | | | | |
| Right-Temporalpole (38), Superior Temporal Gyrus | **4792** | **0.012** | **48570.60** | **38 15 -30** |
| Right-Insula (13) |  | 0.032 | 32939.62 | 33 11 7 |
| Left-InfTempGyrus (20), | **560** | **0.042** | **28909.53** | **-41 -17 -25** |
| Left-Parahipp (36), Uncus |  | 0.049 | 26459.82 | -24 -5 -29 |
| Right-AntPFC (10), Medial Frontal Gyrus | **633** | **0.048** | **27035.82** | **6 52 15** |
| Left-MedTempGyrus (21) | **109** | **0.049** | **26633.61** | **-50 -17 -14** |
| Right-OrbFrontal (11), Rectal Gyrus | **48** | **0.050** | **26356.28** | **5 23 -25** |
| Right-dlPFC(dorsal) (9), Superior Frontal Gyrus | **49** | **0.050** | **26319.98** | **27 46 31** |
| Positive associations of CTH with H5PT, adjusted for RMET (N=80) | | | | |
| Right-FrontEyeFields (8), Middle Frontal Gyrus | **4578** | **0.028** | **32969.95** | **28 11 47** |
| Right-dlPFC(lat) (46), Inferior Frontal Gyrus |  | 0.029 | 32220.45 | 45 35 7 |
| Right-OrbFrontal (11), Medial Frontal Gyrus |  | 0.036 | 29483.57 | 4 45 -18 |
| Results are listed at p < 0.05 FWE_TFCE_ whole-brain corrected with corresponding cluster size equivalent (equivk),  TFCE scores and MNI coordinates. Bold data indicate primary peaks and non-bold data indicate secondary peaks  within a cluster. BA, [Brodmann area](https://www.sciencedirect.com/topics/neuroscience/brodmann-area). *RMET* Reading the Mind in the Eyes Test; *H5PT* Hamasch-Five- Point Test; *sFLU* semantic Fluency; | | | | |

| Table S6: Partial correlations of Reading the Mind in the Eyes and executive functions test performance with gray matter structural covariance networks | | | | | | | | | | | | |
| --- | --- | --- | --- | --- | --- | --- | --- | --- | --- | --- | --- | --- |
|  | **SBM Components** | | | | | | | | | | | |
| Test | **C1** | **C2** | **C3** | **C4** | **C5** | **C6** | **C7** | **C8** | **C9** | **C10** | **C11** | **C12** |
| RMET rho | -0.11 | **0.33** | **0.37** | -0.17 | 0.14 | -0.19 | -0.22 | 0.21 | 0.18 | **0.42** | 0.08 | **0.35** |
| pFDR | n.s. | **0.02** | **0.012** | n.s. | n.s. | n.s. | n.s. | n.s. | n.s. | **0.002** | n.s. | **0.02** |
| H5PT rho | -0.17 | 0.21 | 0.05 | -0.20 | 0.14 | **-0.26** | 0.08 | **0.25** | 0.20 | **0.32** | -0.01 | **0.42** |
| pFDR | n.s. | n.s. | n.s. | n.s. | n.s. | **0.06** | n.s. | **0.08** | n.s. | **0.02** | n.s. | **0.002** |
| sFlu rho | 0.06 | **0.26** | 0.16 | 0.03 | 0.02 | -0.08 | 0.00 | **0.38** | 0.20 | **0.34** | -0.02 | 0.19 |
| pFDR | n.s. | **0.06** | n.s. | n.s. | n.s. | n.s. | n.s. | **0.002** | n.s. | **0.01** | n.s. | n.s. |
| pFlu rho | 0.02 | 0.18 | 0.01 | -0.01 | 0.12 | -0.10 | -0.07 | **0.31** | 0.11 | **0.45** | -0.03 | **0.29** |
| pFDR | n.s. | n.s. | n.s. | n.s. | n.s. | n.s. | n.s. | **0.02** | n.s. | **0.002** | n.s. | **0.04** |
| TMT rho | 0.11 | -0.22 | 0.10 | **0.32** | -0.14 | -0.03 | -0.05 | -0.14 | -0.12 | -0.21 | -0.02 | -0.28 |
| pFDR | n.s. | n.s. | n.s. | **0.06** | n.s. | n.s. | n.s. | n.s. | n.s. | n.s. | n.s. | n.s. |
| Stroop rho | -0.08 | 0.18 | -0.16 | -0.16 | 0.18 | -0.16 | 0.05 | **0.31** | 0.11 | **0.35** | 0.08 | 0.23 |
| pFDR | n.s. | n.s. | n.s. | n.s. | n.s. | n.s. | n.s. | **0.05** | n.s. | **0.02** | n.s. | n.s. |
| Partial spearman correlations (rho) of structural covariance network loading coefficients with RMET (N=80) and EF (i.e.: H5PT N=89; sFLU N=95; pFLU N=92; TMT N=64; Stroop N=72) test performance (all partial spearman correlations adjusted for age and gender). Results are listed at p<0.1 False Discovery Rate (FDR) corrected; *n.s.* not statistically signignificant. *RMET* Reading the Mind in the Eyes Test; *H5PT* Hamasch-Five- Point Test; *sFLU* semantic Fluency; *pFLU* phonemic Fluency; *TMT* Trail Making Test B/A ratio (with lower scores indicating better performance); | | | | | | | | | | | | |
