# Supplementary material for: Dissecting neural correlates of theory of mind and executive functions in behavioral variant frontotemporal dementia
Source: Alzheimers Res Ther. 2024 Oct 26;16:237. doi: 10.1186/s13195-024-01596-4 (PMC11515257; doi:10.1186/s13195-024-01596-4)
Supplement: Supplementary file 3 — Supplementary Material 3. [file 13195_2024_1596_MOESM3_ESM.pdf]

Table of Contents

 1
SBM ICA Parameters 1
ICASSO Plots 2
SBM components 2
PCA components variance summary 13
ICA components variance summary 14
Kurtosis of loading coefficients and spatial maps 14
FNC correlations of subject loadings 16
FNC metrics of component spatial maps 17

SBM ICA Parameters

...

Number of Independent Components : 12

ICA Algorithm : Infomax

Number Of Subjects : 103

Mask File : Default Mask Created From Structural Data

PCA Type : Standard

Scaling Components : Z-scores

Stability analysis type : none

Anatomical file: /Users/christopherweise/Desktop/gift-master/GroupICAT/icatb/icatb_templates/ch2bet_3x3x3.nii
Slice Plane: Axial

Image values: Positive and negative

Convert to Z-scores: yes

Threshold: 2

...

ICASSO Plots

SBM components

Group spatial maps and subject loading coefficients

- **a) Subject loading coefficients**
- **b) Group spatial maps**

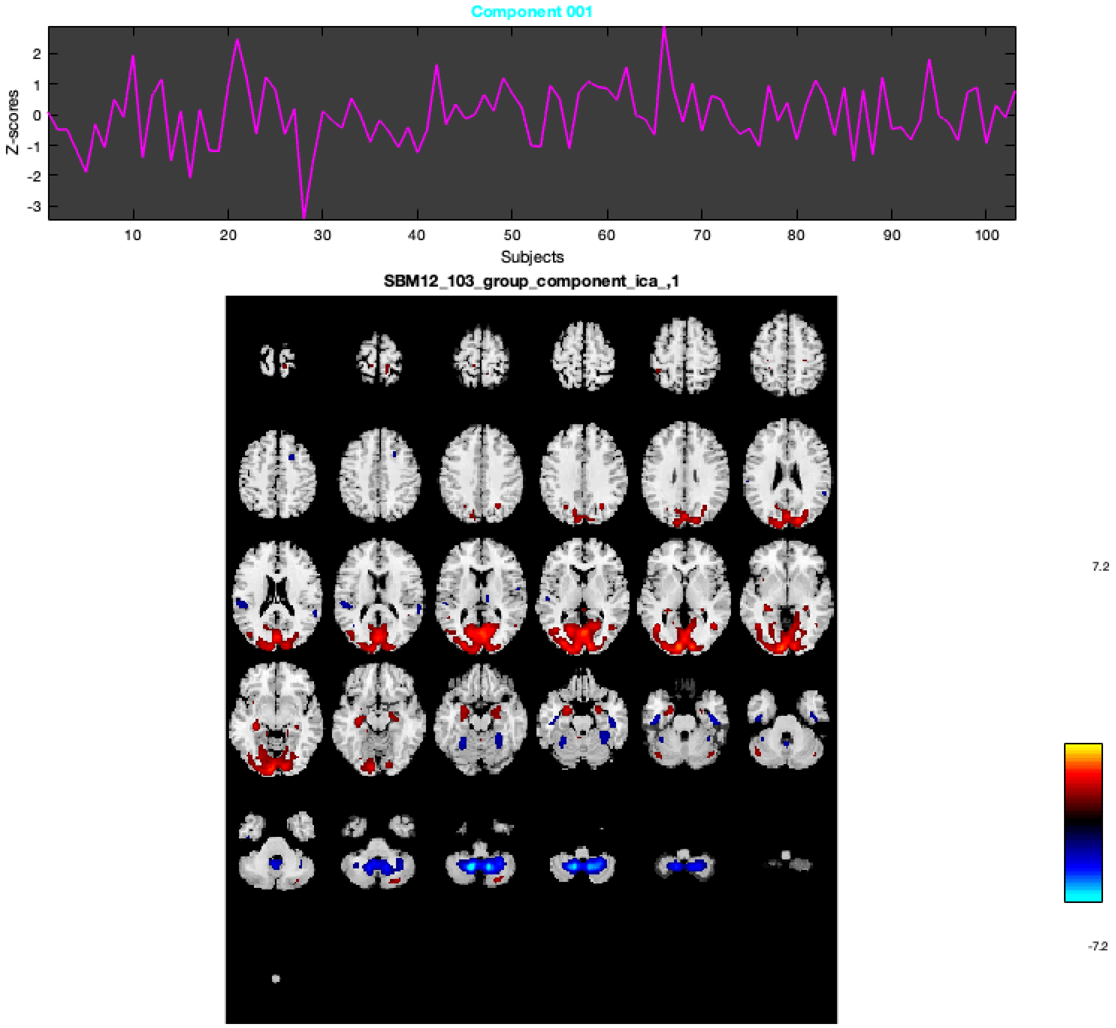

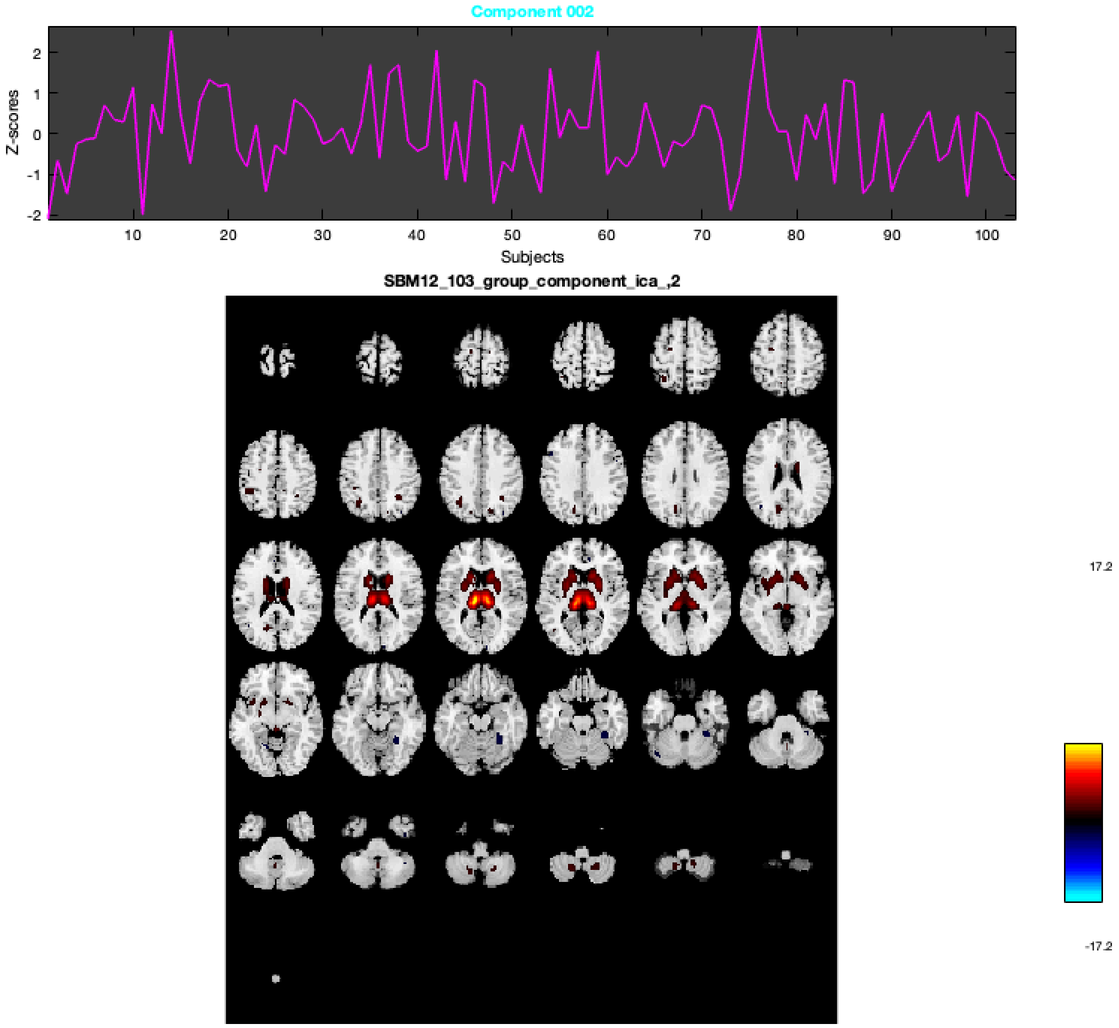
Component 002
Z-scores
2
1
0
-1
-2
10
20
30
40
50
60
70
80
90
100
Subjects
SBM12_103_group_component_ica_,2
17.2
-17.2

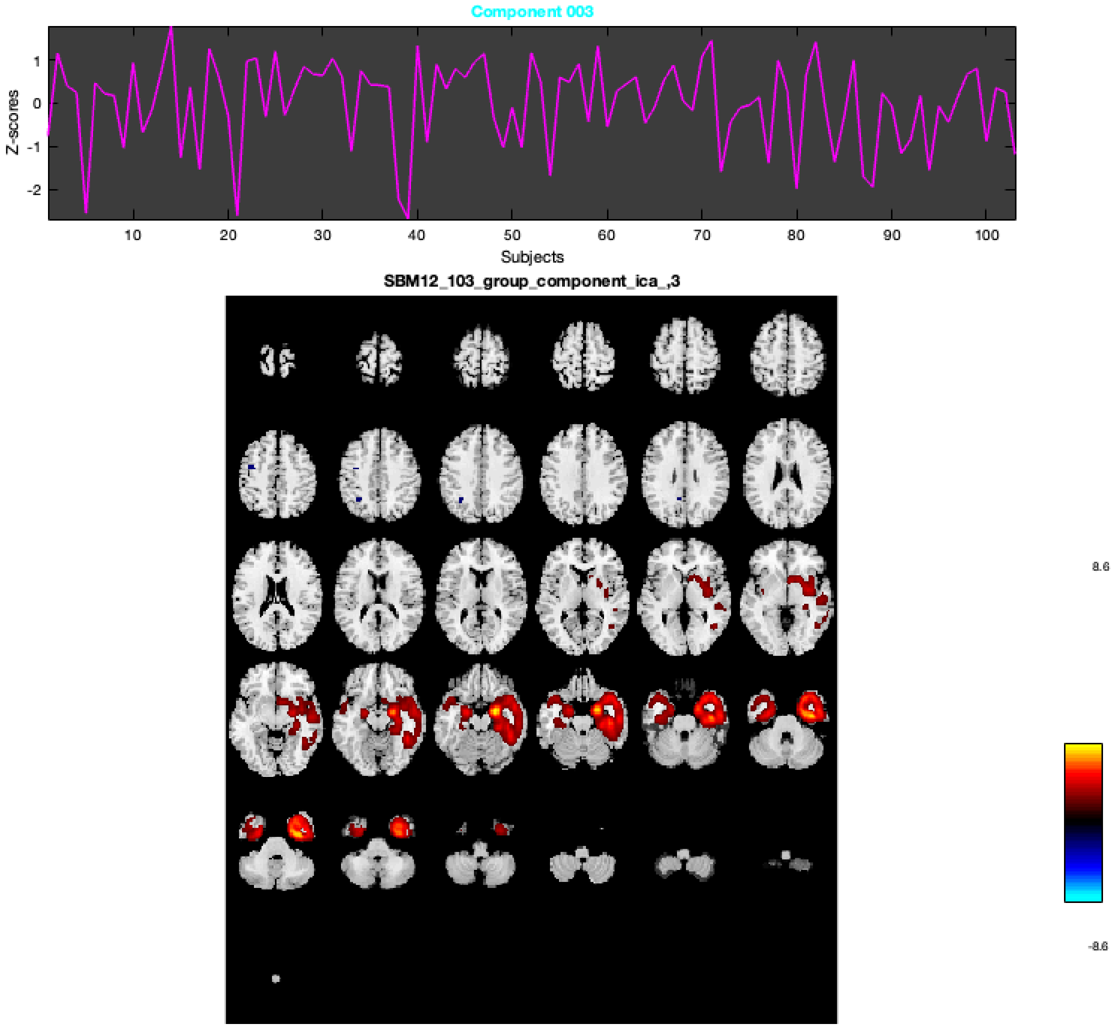
Component 003
Z-scores
Subjects
SBM12_103_group_component_ica_,3
8.6
-8.6

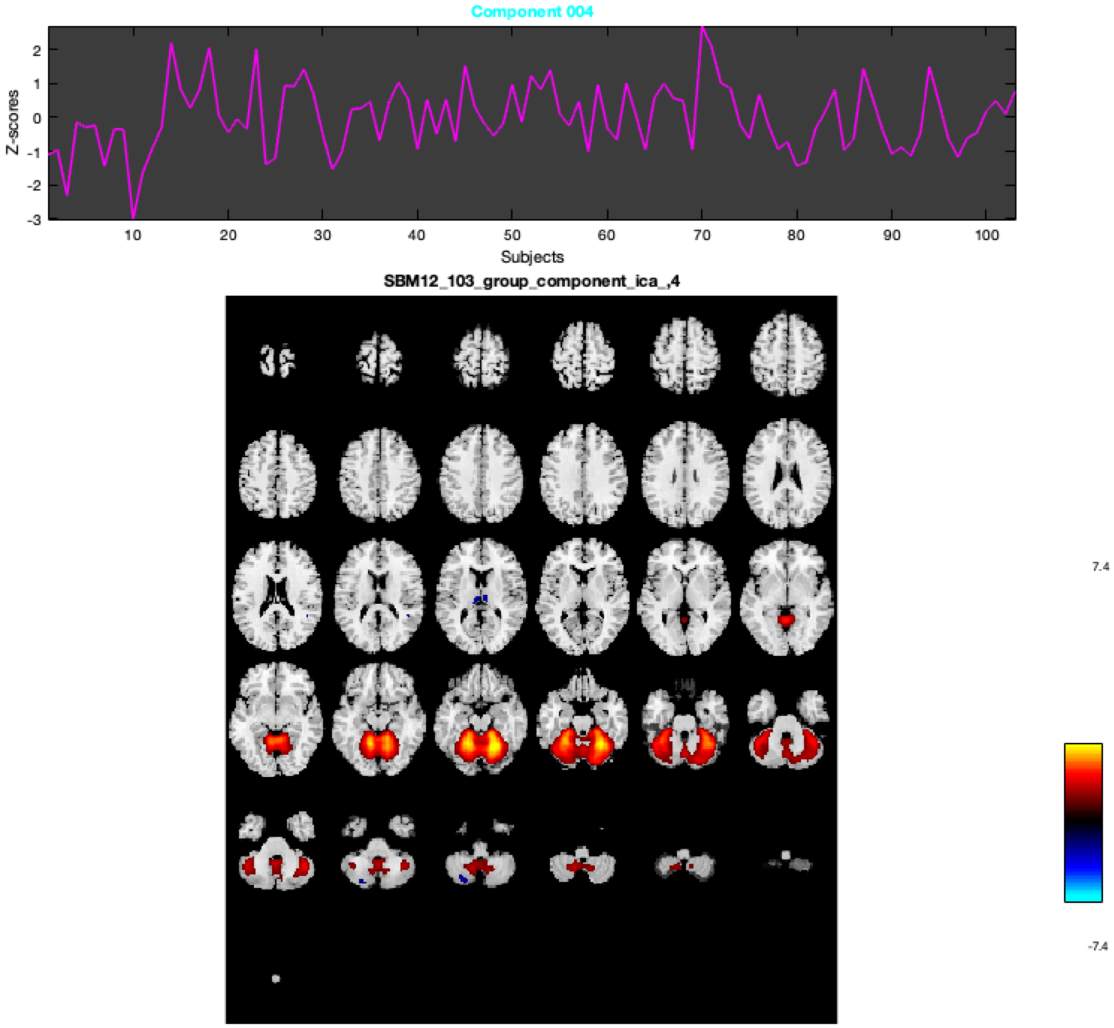
Component 004
Z-scores
Subjects
2
1
0
-1
-2
-3
10
20
30
40
50
60
70
80
90
100
SBM12_103_group_component_ica_,4
7.4
-7.4

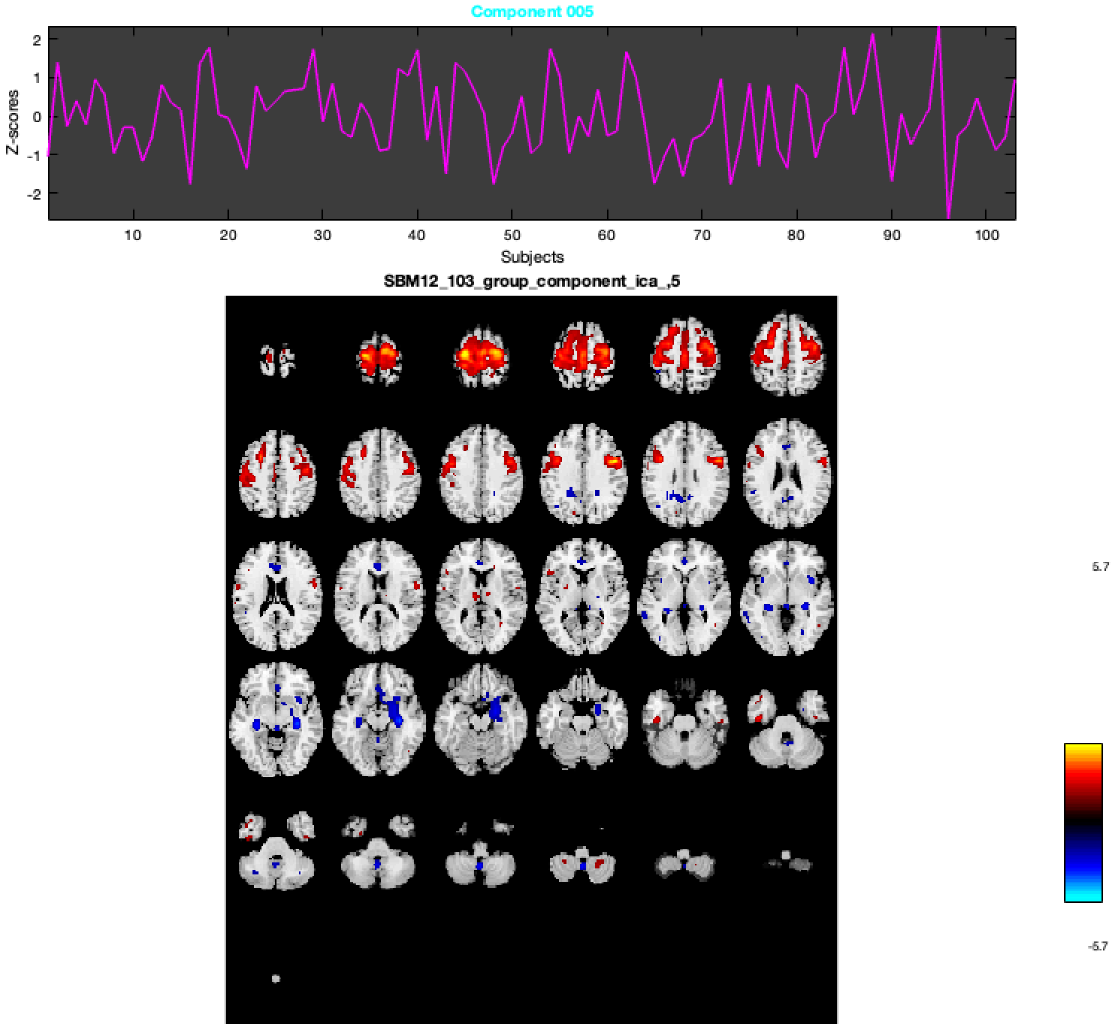
Component 005
Z-scores
Subjects
SBM12_103_group_component_ica_,5
5.7
-5.7

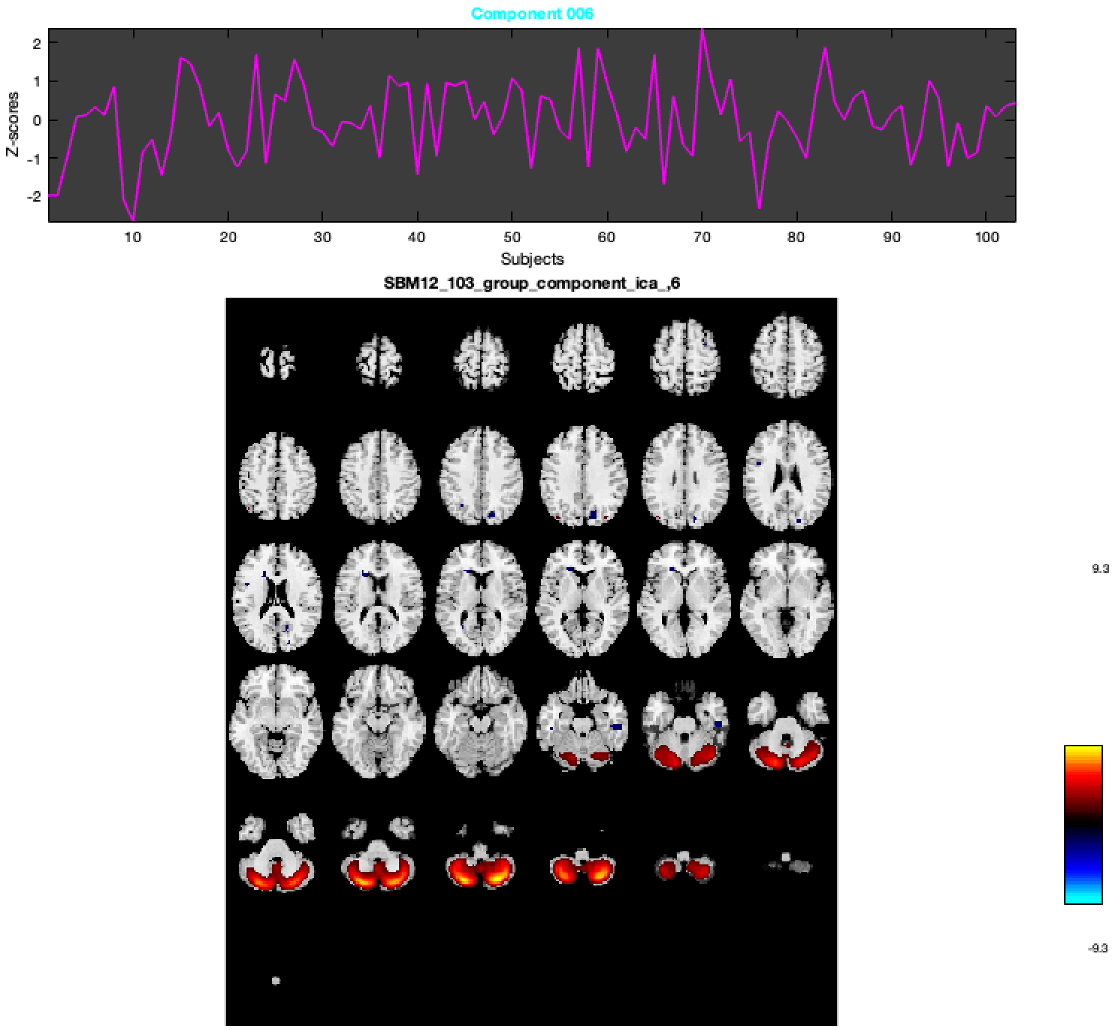
Component 006
Z-scores
Subjects
SBM12_103_group_component_ica_,6
9.3
-9.3

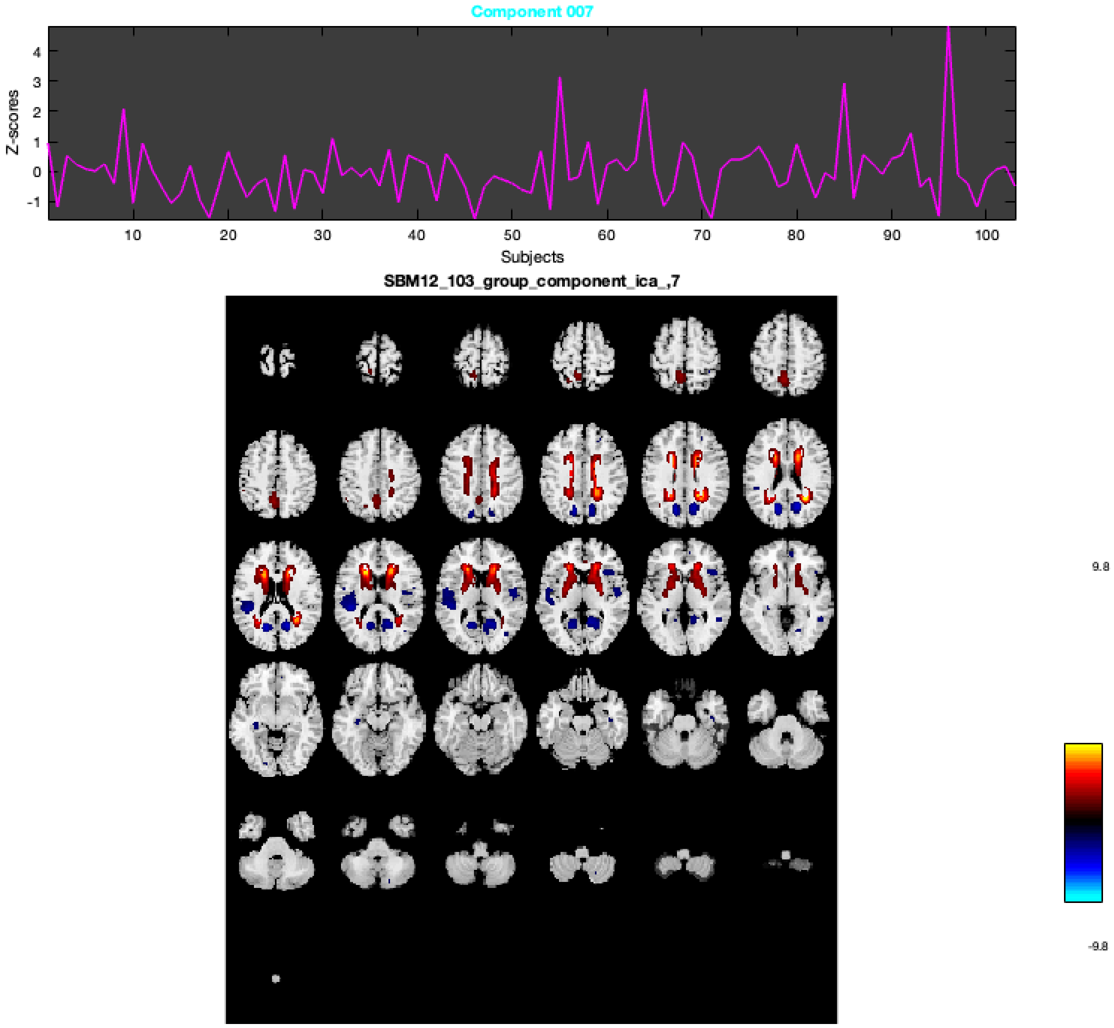
Component 007
Z-scores
4
3
2
1
0
-1
10
20
30
40
50
60
70
80
90
100
Subjects
SBM12_103_group_component_ica_,7
9.8
-9.8

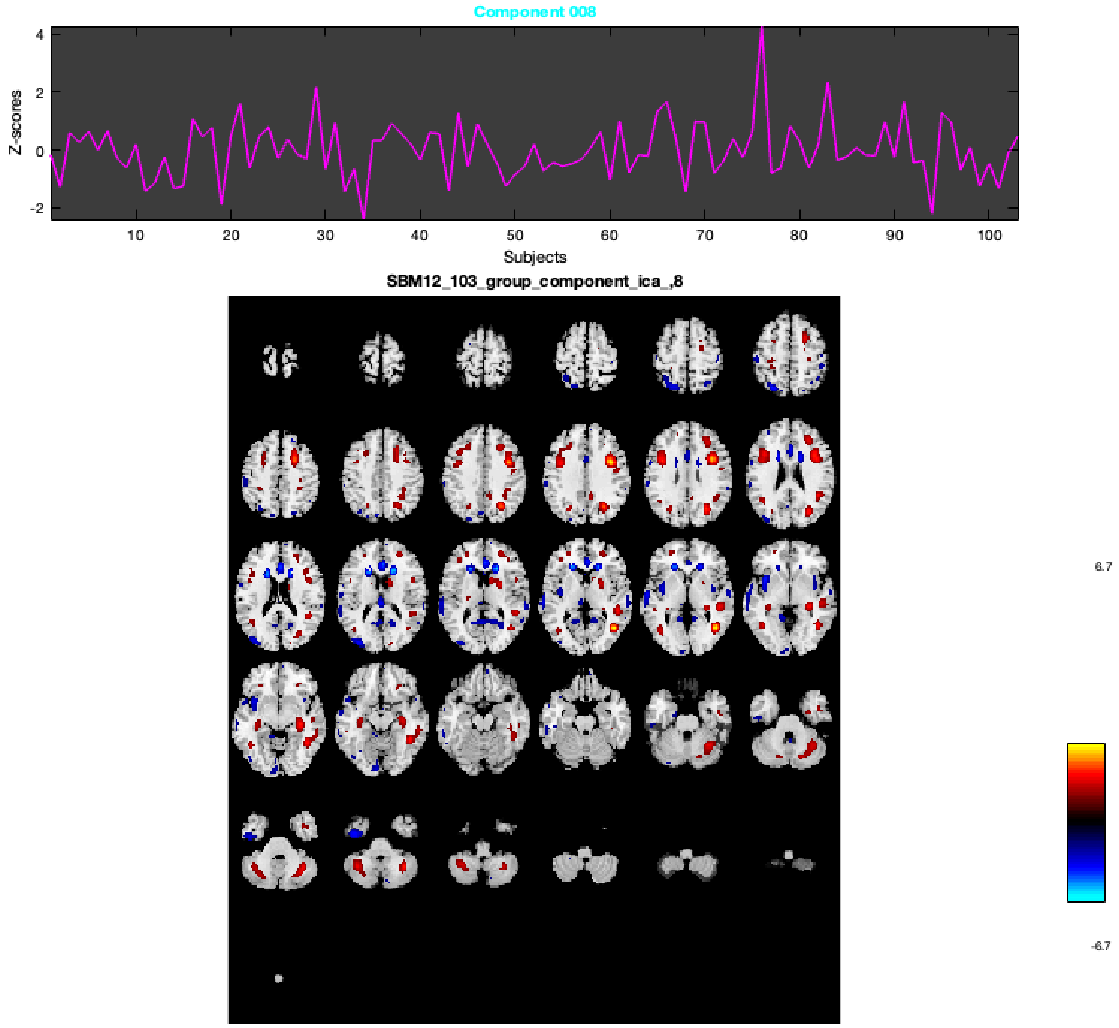
Component 008
Z-scores
Subjects
SBM12_103_group_component_ica_,8
6.7
-6.7

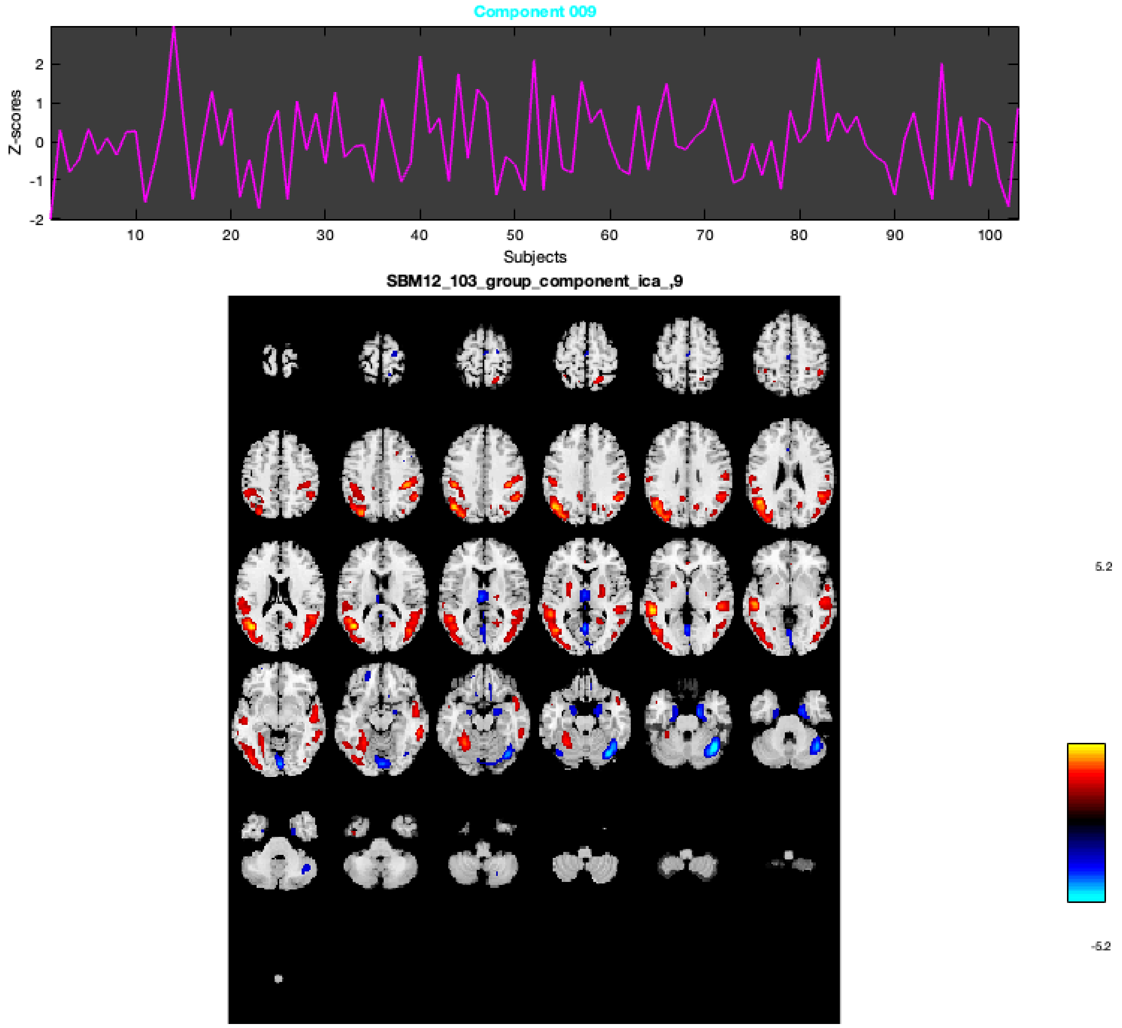
Component 009
Z-scores
2
1
0
-1
-2
10
20
30
40
50
60
70
80
90
100
Subjects
SBM12_103_group_component_ica_,9
5.2
-5.2

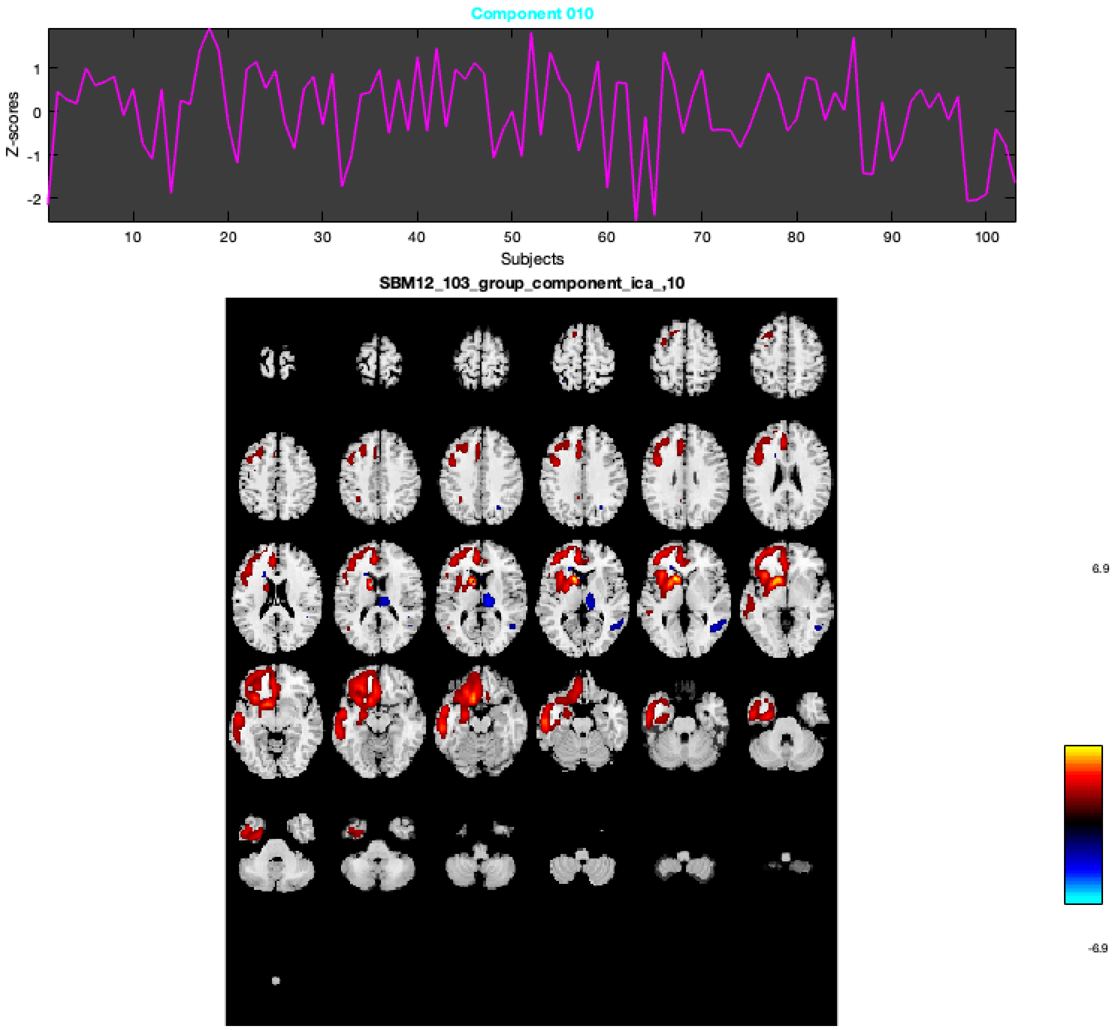
Component 010
Z-scores
Subjects
SBM12_103_group_component_ica_,10
6.9
-6.9

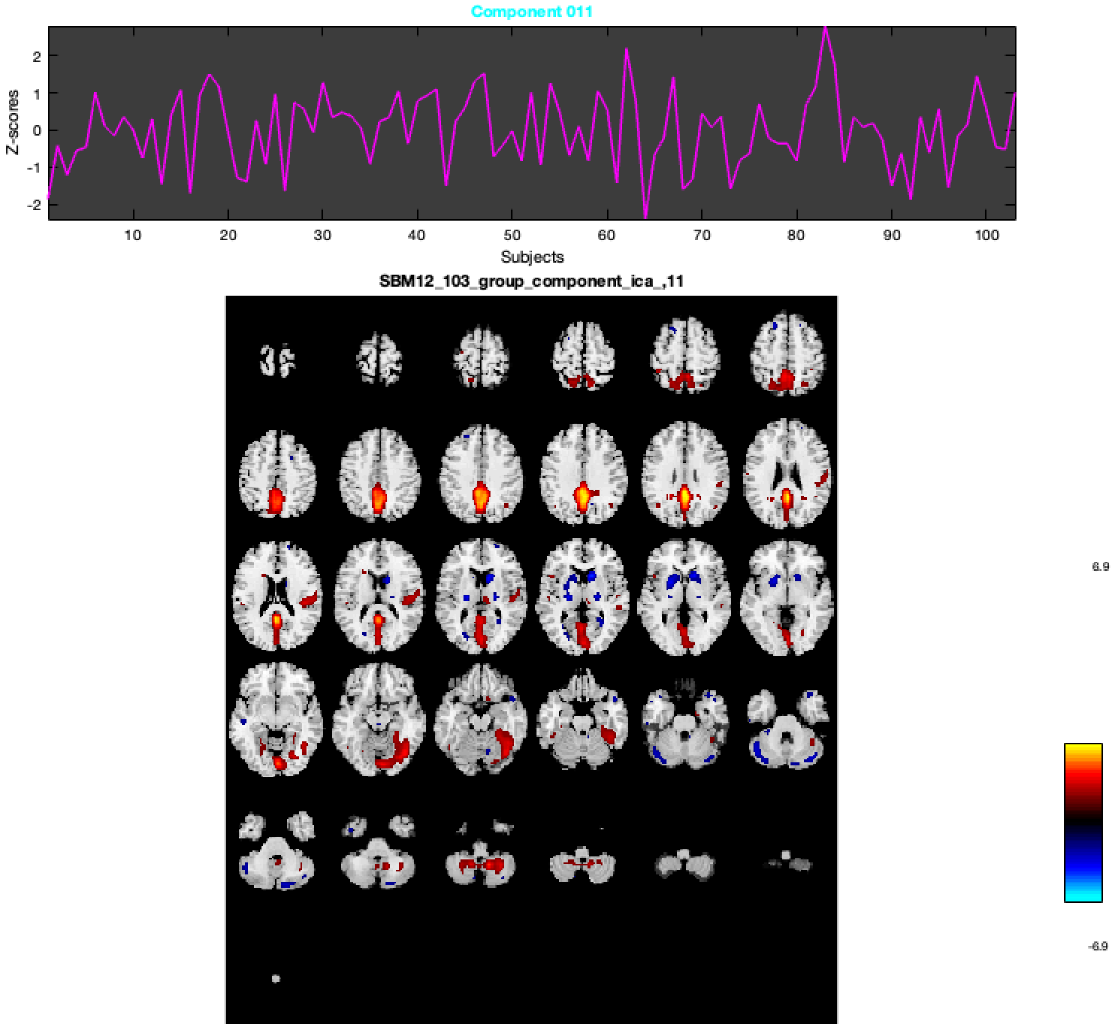
Component 011
Z-scores
Subjects
SBM12_103_group_component_ica_,11
6.9
-6.9

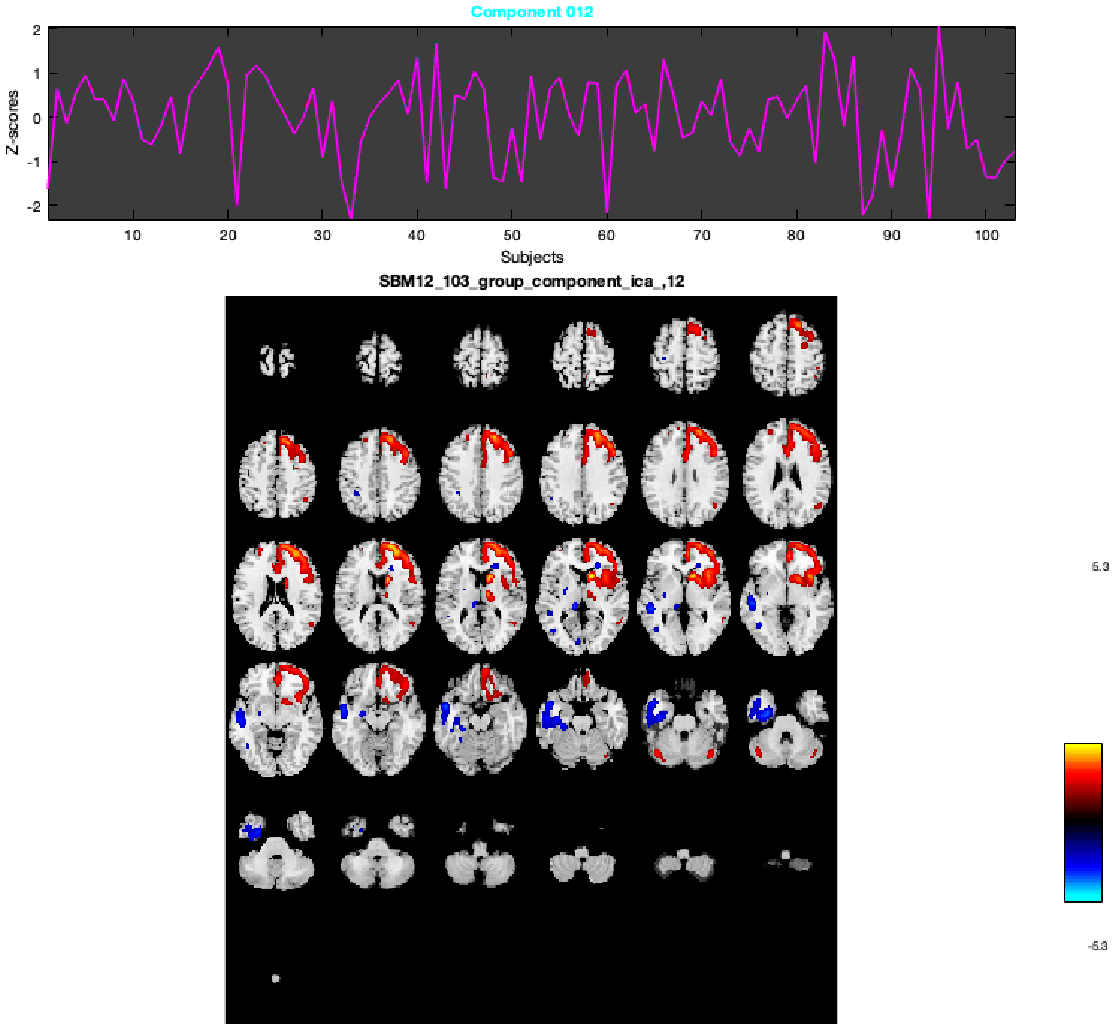

PCA components variance summary

- **Percent variance** - Percent variance is reported for each component.

```
ComponentNumber    PercentVariance
_______________    _______________

       1               0.21609
       2               0.21979
       3               0.23822
       4               0.24392
       5                0.3207
       6               0.34146
       7                0.4206
```

8	0.48353
9	0.74819
10	1.063
11	1.806
12	87.904

ICA components variance summary

- **Percent variance** - Percent variance is reported for each component.

ComponentNumber	PercentVariance
1	1.3467
2	4.5252
3	8.0356
4	16.555
5	3.2609
6	6.731
7	2.6902
8	0.3367
9	7.6916
10	8.5169
11	10.917
12	7.1864

Kurtosis of loading coefficients and spatial maps

ComponentNumber	Timecourses	SpatialMaps
1	3.7556	8.1348
2	2.8228	46.165
3	2.9807	13.895
4	3.199	15.307
5	2.6129	5.593
6	2.8378	21.337
7	8.4982	15.385
8	5.3527	5.3457
9	2.879	5.1237
10	2.7313	7.0979
11	2.6773	8.089
12	2.5762	5.2619

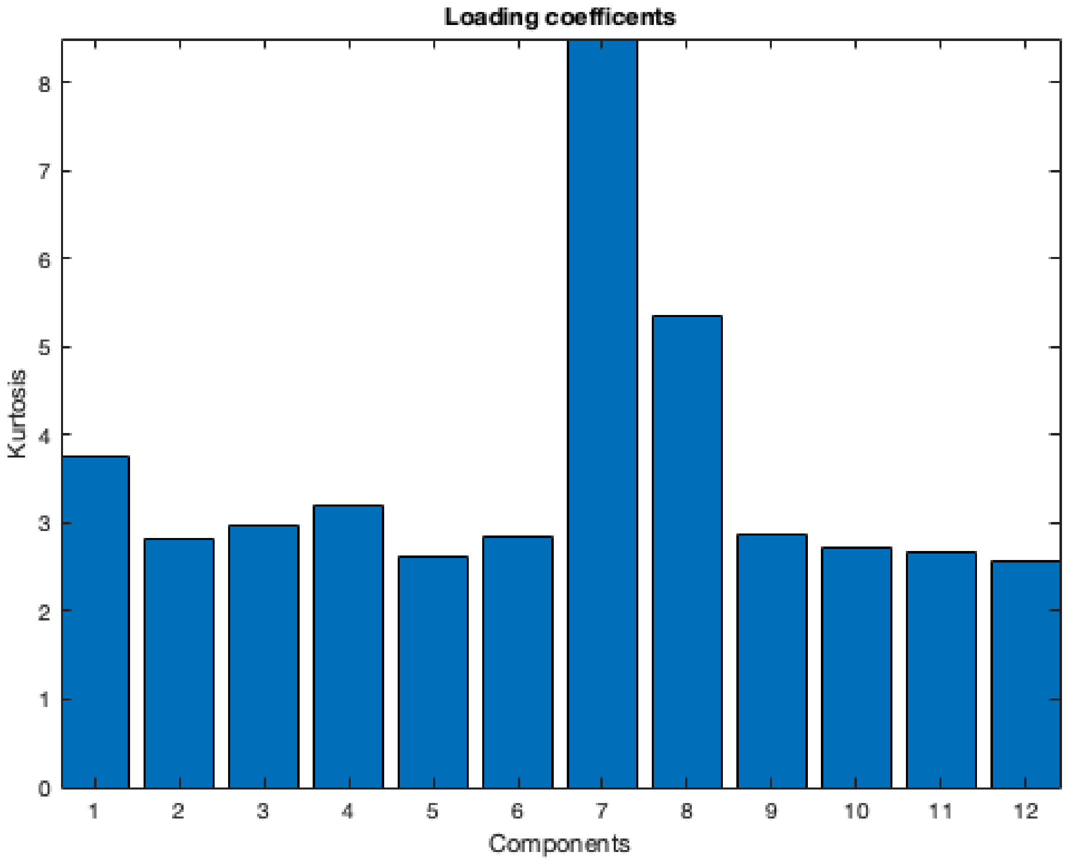
Loading coefficents
Kurtosis
0
1
2
3
4
5
6
7
8
1
2
3
4
5
6
7
8
9
10
11
12
Components

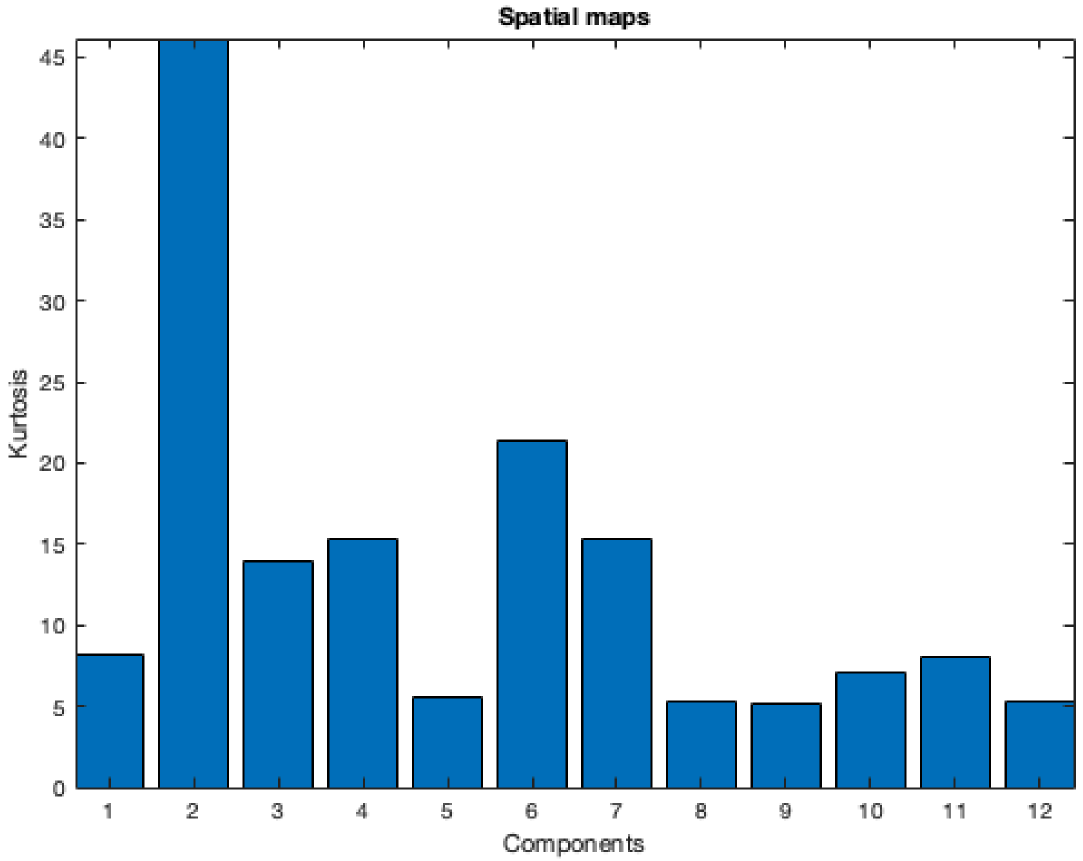

FNC correlations of subject loadings

Functional network connectivity correlations

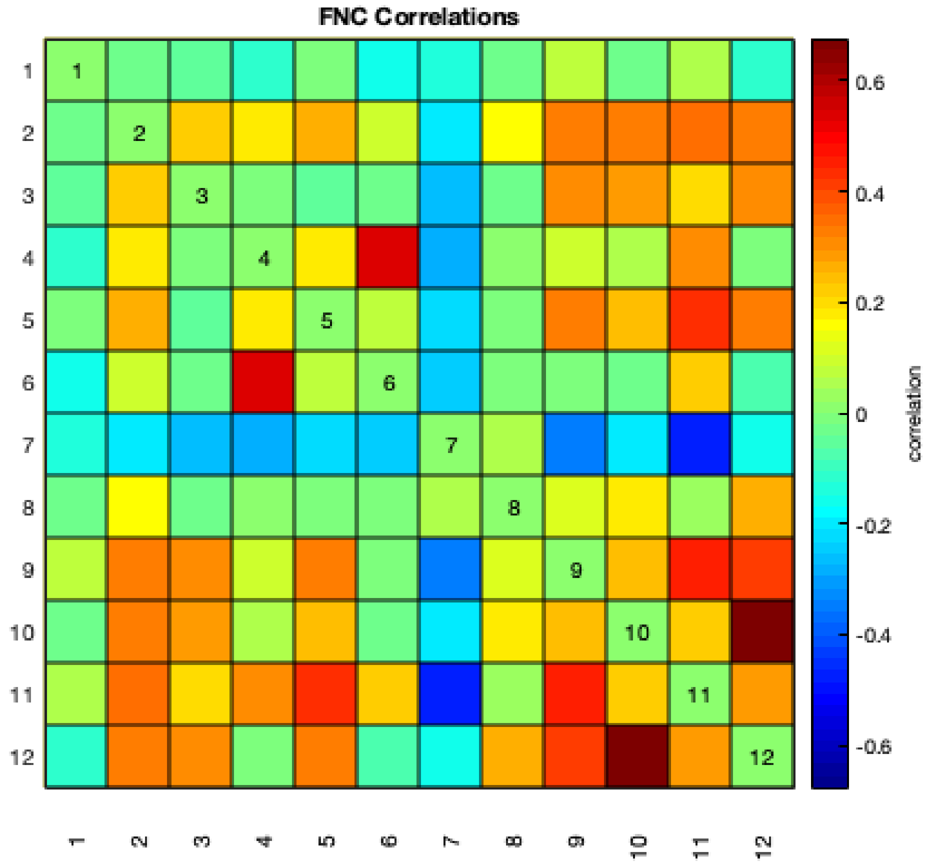

FNC metrics of component spatial maps

Mutual information is computed between components spatially

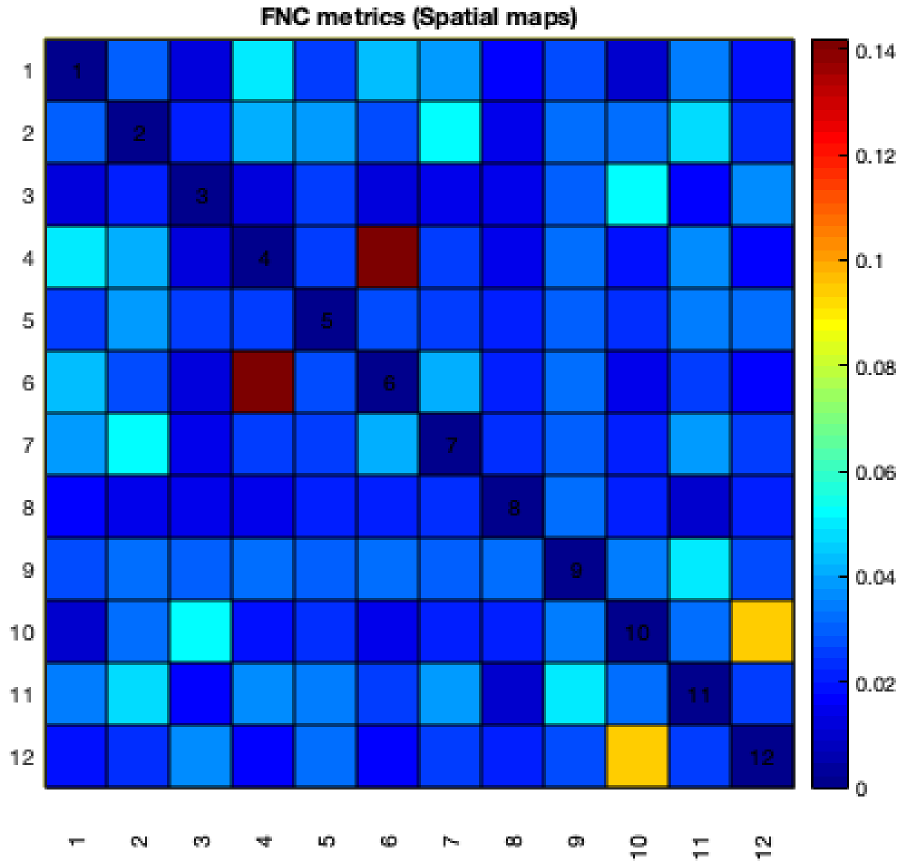

Published with MATLAB® R2023a
